# Chiroptical Second-Harmonic Tyndall Scattering from Silicon Nanohelices

**DOI:** 10.1021/acsnano.4c02006

**Published:** 2024-06-17

**Authors:** Ben J. Olohan, Emilija Petronijevic, Ufuk Kilic, Shawn Wimer, Matthew Hilfiker, Mathias Schubert, Christos Argyropoulos, Eva Schubert, Samuel R. Clowes, G. Dan Pantoş, David L. Andrews, Ventsislav K. Valev

**Affiliations:** † Centre of Photonics and Photonic Materials, University of Bath, Bath BA2 7AY, U.K.; ‡ Centre of Nanoscience and Nanotechnology, University of Bath, Bath BA2 7AY, U.K.; § SBAI Department, La Sapienza University of Rome, Rome 00161, Italy; ∥ Department of Electrical and Computer Engineering, University of Nebraska-Lincoln, Lincoln, Nebraska 68588, United States; ⊥ Solid State Physics and NanoLund, Lund University, Box 118, Lund, Skane 22100, Sweden; # Department of Electrical Engineering, The Pennsylvania State University, University Park, Pennsylvania 16803, United States; ¶ Department of Chemistry, University of Bath, Bath BA2 7AY, U.K.; ∇ Centre for Photonics and Quantum Science, University of East Anglia, Norwich NR4 7TJ, U.K.

**Keywords:** nanophotonics, chirality, nanoparticles, nanomaterials, metamaterials

## Abstract

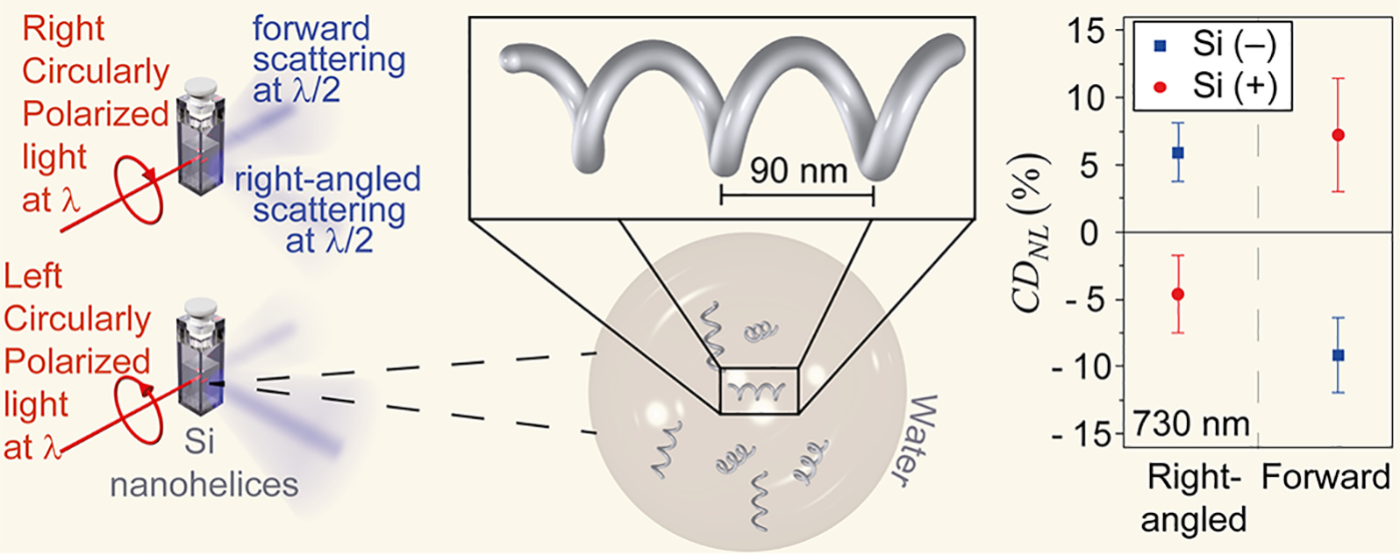

Chirality is omnipresent in the living world. As biomimetic nanotechnology and self-assembly advance, they too need chirality. Accordingly, there is a pressing need to develop general methods to characterize chiral building blocks at the nanoscale in liquids such as water—the medium of life. Here, we demonstrate the chiroptical second-harmonic Tyndall scattering effect. The effect was observed in Si nanohelices, an example of a high-refractive-index dielectric nanomaterial. For three wavelengths of illumination, we observe a clear difference in the second-harmonic scattered light that depends on the chirality of the nanohelices and the handedness of circularly polarized light. Importantly, we provide a theoretical analysis that explains the origin of the effect and its direction dependence, resulting from different specific contributions of “electric dipole–magnetic dipole” and “electric dipole–electric quadrupole” coupling tensors. Using numerical simulations, we narrow down the number of such terms to 8 in forward scattering and to a single one in right-angled scattering. For chiral scatterers such as high-refractive-index dielectric nanoparticles, our findings expand the Tyndall scattering regime to nonlinear optics. Moreover, our theory can be broadened and adapted to further classes where such scattering has already been observed or is yet to be observed.

## Introduction

Chiral inorganic nanoparticles dispersed in liquids have shown potential for various technological applications^1^ including sensing,^2^ therapeutics, catalysis, chiral separation,^3^ nanorobotics,^4^ etc. Chiral nanoparticles are useful in the development of sensitive chiral sensors and especially biosensors.^5^ Dispersing these nanoparticles in a liquid medium allows them to interact with chiral molecules, for instance, to form hybrid materials.^6^ As a result, their optical, electrical, and magnetic properties change. In turn, these changes serve to detect chiral analytes. Chiral nanoparticles can also be incorporated into liquid-based drug delivery systems for therapeutic purposes, e.g., by triggering an immune response,^7^ selectively cutting DNA,^8^ killing viruses,^9^ or targeting peptides associated with Alzheimer’s disease.^10^ These studies are emblematic of the emerging field of biomimetic therapeutics. Moreover, chiral inorganic nanoparticles dispersed in liquid media can act as catalysts in various chemical reactions;^11^ for instance, they can enable photocatalysts to cleave proteins depending on the handedness of circularly polarized illumination.^12^ Additionally, chiral nanoparticles dispersed in liquid matrices can be employed in the development of advanced optical devices. The interactions between light and these nanoparticles lead to chiroptical effects such as circular dichroism (CD) and optical rotation. These effects could play a role in optical filters,^13^ circularly polarized light sources,^14^ polarizers, and other photonic devices for applications ranging from telecommunications to displays. For all the above-mentioned applications, a key question is how to accurately characterize the chirality within liquids, at the nanoscale?

Recently, chiroptical harmonic scattering (CHS) has emerged as a nonlinear optical technique capable of characterizing the chirality of nanoparticles, freely revolving in an isotropic liquid environment.^15^ This technique can probe minuscule volumes of illumination; it has been demonstrated in a liquid volume of 1 μL,^16^ wherein the volume of actual light–matter interaction was estimated at 40 μm^3^.^17^ Moreover, the technique is highly sensitive, with average sensitivity down to the single nanoparticle level.^17,18^ In plasmonic nanomaterials, CHS has been observed in Rayleigh scattering at both the second- and third-harmonic frequency.^19^ We note that second- and third-harmonic nonlinear effects are fundamentally different, as they depend on different material properties and different selection rules.^20,21^ Second-harmonic techniques^22^ are often associated with surface/interface responses that can be down to single atomic levels,^23^ whereas third-harmonic emission is typically allowed from the whole volume of light penetration inside a material.

In semiconducting nanomaterials, CHS has only been observed at the third-harmonic frequency of illumination. Moreover, it was only observed in Mie scattering, where the size of the particles was >3 times larger than the wavelength of illumination, which leads to interference between light scattering from different regions of the same particles and causes a more complex scattering pattern (compared to Rayleigh scattering). Additionally, it was only observed from the direct band gap semiconductor CdTe. By contrast, indirect band gap materials (such as silicon) have more restricted efficiency in processes involving light emission or absorption. This is because indirect band gap semiconductors typically require additional mechanisms, such as phonon interactions to facilitate transitions between the valence and conduction bands. In this context, indirect band gap materials behave more like “high-refractive-index dielectrics” (e.g., semiconductors with very low value of their loss tangent). Recently, high-refractive-index dielectrics have emerged as a promising class of optical materials that can sustain Mie resonances and other resonating effects.^24^

Due to their contrast to the surrounding media, high-refractive-index dielectric nanomaterials benefit from enhanced light–matter interactions. Such nanomaterials have found applications in biosensing,^25,26^ stimulated Raman scattering,^27^ holography,^28−30^ color printing,^31^ and encryption.^32^ Among the high-refractive-index dielectrics, silicon (Si) stands out for its versatility, wide availability, and high compatibility with existing electronics technology platforms. Si has been used in the context of drug delivery,^33^ optical trapping in solution,^34^ and chirality.^35,36^ In nonlinear optics, enhanced third-^37,38^ and second-harmonic^39,40^ responses have also been reported based on Si nanostructures. Evidently, high-refractive-index nanomaterials represent a promising emergent class of photonic materials, but so far, they have remained inaccessible to CHS.

Here, we present the chiroptical second-harmonic Tyndall scattering effect,^41−45^ from Si nanohelices (∼270 nm long). Specifically, the nanohelices were dispersed in water and illuminated with wavelengths (λ) in the range of 710–750 nm. Upon illumination with circularly polarized light, we report a difference in scattered second-harmonic intensity that depends on the chirality of the nanohelices and of the light. The result is observed both in forward scattering and in right-angled scattering (with respect to the incident wavevector) at three different wavelengths. Our nanohelices are fabricated from a high-refractive-index material,^46^ whose properties in the visible are almost dielectric (see Supporting Information and Figure S1). We employ a combination of analytical theory and electromagnetic simulations to identify the origin of the second-harmonic scattering chiroptical response. Depending on the direction of scattered light, we also identify specific contributions from “electric dipole–magnetic dipole” and “electric dipole–electric quadrupole” coupling tensors. Accordingly, the sign of the chiroptical response can change depending on the direction of light scattering, in agreement with our experimental results.

## Results and Discussion

**Figure 1:**
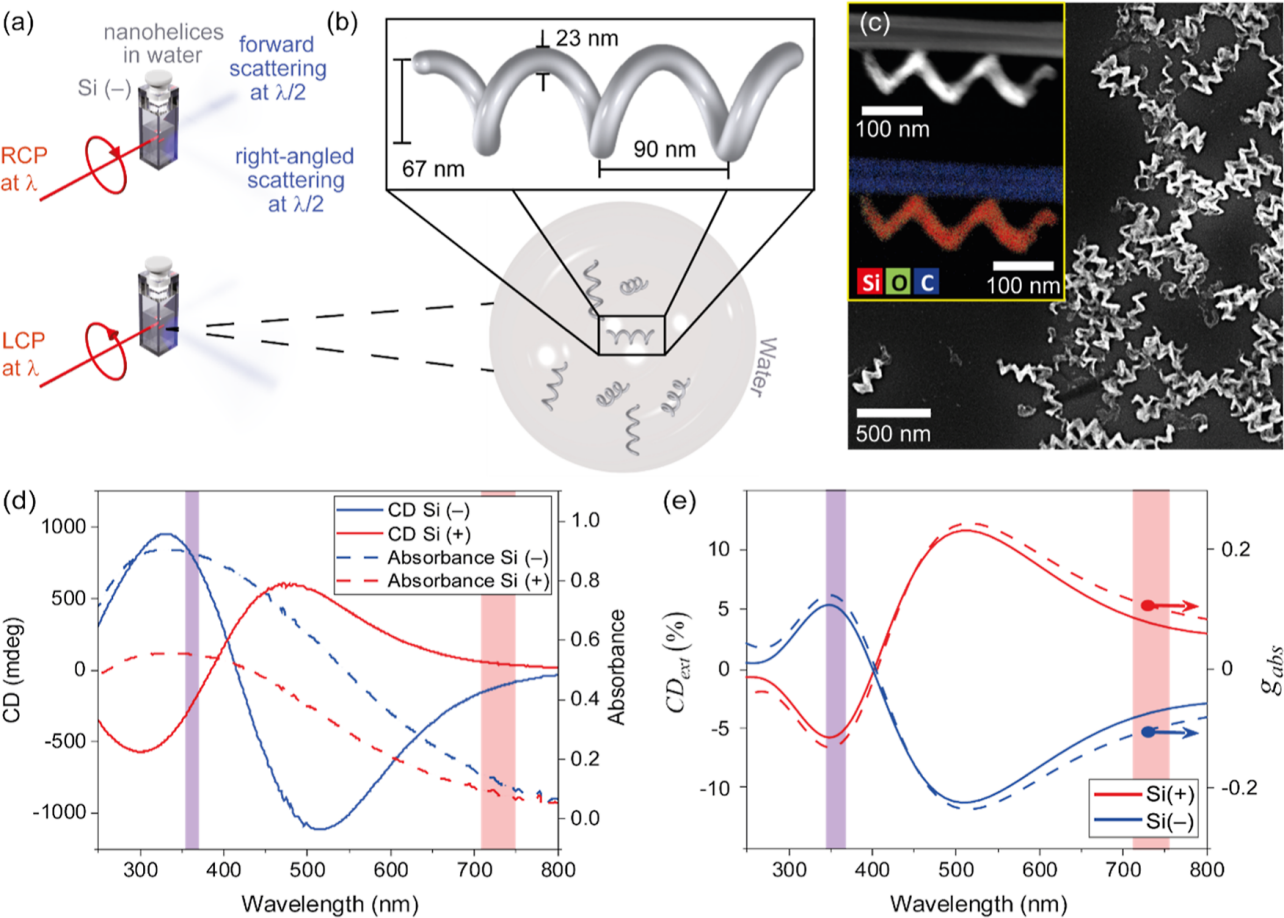
Si nanohelices exhibit a strong chiroptical response across the spectrum, with pronounced absorbance that peaks in the UV and trails in the near-infrared spectrum. (a) Diagram showing second-harmonic scattering optical activity. Upon illuminating a suspension of Si nanohelices, with RCP or LCP light, respectively, at wavelength λ, the intensity of light scattered at wavelength λ/2 changes for both forward and right-angled scattering. The intensity of scattered light in this diagram is not to scale. (b) Average dimensions of the nanohelices that are randomly dispersed in water for our measurements. (c) High-resolution SEM and element-specific imaging of the nanohelices. (d) CD measurements in the linear optical regime of the two enantiomorphs of Si nanohelices: Si (−) is colored blue, and Si(+) is colored red (full lines). The corresponding absorbance spectra peak at ∼350 nm (dashed lines). (e) Numerical simulations of the extinction CD (in full lines) and the dissymmetry factor *g*_abs_ (in dashed lines) of Si(−) in blue and Si(+) in red.

Figure 1a shows a diagram of chiroptical second-harmonic scattering from the Si nanohelices. LCP or RCP light with a frequency ω (wavelength λ) is incident on a cuvette containing Si nanohelices suspended in water; samples were prepared from Si nanohelices deposited on a solid state substrate; see Methods, Supporting Information and Figure S2. Two photons with the same fundamental frequency interact with the nanohelices and one photon with a frequency 2ω (wavelength λ/2) is scattered from the nanohelices. The intensity of the scattered light depends on whether LCP or RCP light is incident and on the handedness of the nanohelices. Figure 1b presents the dimensions of the nanohelices (height 270 nm, pitch 90 nm, wire radius 11 nm, and loop radius 33 nm). These dimensions were obtained from scanning electron microscopy (SEM) image analysis in which 600 measurements were averaged. An SEM image of a nanohelix is shown in Figure 1c that also provides the elemental composition of the helix. Evidently, the helix is almost entirely composed of silicon (shown in red), with only small amounts of oxygen (green). As demonstrated by the absorbance spectra in Figure 1d, the nanohelices exhibit a broad electromagnetic resonance band that peaks in the UV and covers the entire visible spectrum. Within this spectral range, dichroic interactions with circularly polarized light are especially strong, reaching up to 1000 mdeg, as demonstrated by the bisignate CD spectra for the two chiral forms (enantiomorphs), designated Si(+) and Si(−). We note that there is a pronounced CD response in the 300–350 nm spectral range and around 500 nm. The CD spectra are clear mirror images of each other, with the CD from Si(+) samples being only slightly blue-shifted in comparison to the CD from Si(−). The small differences in amplitude and peak position between the two CD spectra are attributed to size variation between the two enantiomorphs; Si(+) and Si(−) are fabricated in different batches. Additionally, there is a difference in concentration between the measured samples; that is due to our fabrication procedure.

To understand the electromagnetic behavior of these nanohelices, we model them by means of finite-difference-time-domain (FDTD) simulations; see Methods and Supporting Information. Since the nanohelices are randomly oriented in water, we have calculated absorption and scattering cross section for different orientations of the nanohelix and performed their orientational average, for LCP and RCP excitations. To compare our simulation results with the experiments, we calculated the normalized extinction difference (CD in extinction) as
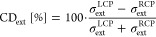
1

Recognizing that in some communities, it is customary to discuss the dissymmetry factor *g*_*i*_, we also define
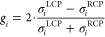
2where *i* = abs and *i* = sc correspond to *g* factor in absorption and scattering, respectively. Figure 1e shows that both CD_ext_ and *g*_abs_ factor correspond very well to the linear experimental CD measurements in Figure 1d. For the Si(+) sample, *g*_abs_ reaches maximum 0.25 and minimum −0.13 values, at the wavelengths of 518 and 350 nm, respectively.

**Figure 2:**
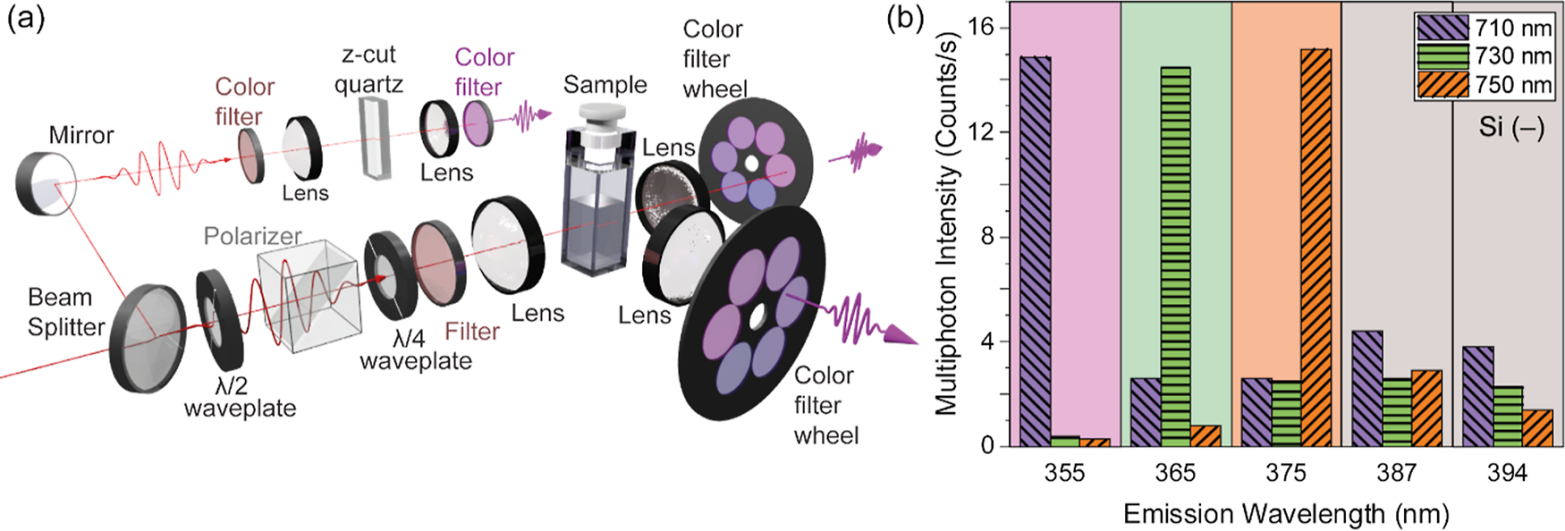
Our experimental setup measures a strong second-harmonic Tyndall scattering signal from the Si nanohelices. (a) Diagram of the experimental setup. The incident laser power is regulated with a combination of a half-wave plate (λ/2) and a polarizer. The direction of circularly polarized light is set with a quarter-wave plate (λ/4). (b) Multiphoton emission intensity at five different wavelengths, upon illumination of the Si(−) nanohelices with 710, 730, and 750 nm. In each case, the second-harmonic emission is very well-resolved above the background multiphoton emission.

To investigate the second-harmonic scattering of Si nanohelices, ultrafast (100 fs) laser pulses (wavelengths of 710, 730, and 750 nm) were first directed through a high-extinction ratio polarizer; see Figure 2a. The well-defined linear polarization state was then converted to circular polarization, using an achromatic quarter-wave plate. Subsequently, a color filter removed any residual second-harmonic light and an aspherical, achromatic, antireflection lens was used to focus the laser into a cuvette holding 1 mL of nanohelices suspended in water. Two lenses were then positioned to collect the second-harmonic scattered light, in the forward direction and at a right angle to the incident beam. After filtering light at the fundamental wavelength, the second-harmonic scattered light was recorded with a photomultiplier tube (PMT). With the laser repetition rate of 80 MHz, the effect of any short-term laser peak-power fluctuations was minimized by averaging the counts over 5 s. The effects of long-term laser power fluctuations were mitigated by implementing a reference line to the setup where the second-harmonic signal from *z*-cut quartz was recorded at the beginning and at the end of each series of measurements. These data were then compared to a laser power calibration curve. Further details on the experimental setup are included in the Methods section.

The Si nanohelices emit a distinct second-harmonic scattering signal that is well above the multiphoton emission background. Figure 2b presents the multiphoton emission intensity at 5 different wavelengths, for 3 separate wavelengths of illumination, i.e., 710 (purple bar with lines from upper left to lower right), 730 (green bar with horizontal lines), and 750 nm (orange bar with lines from lower left to upper right). In each case, the fundamental laser power was 7 mW and the polarization state was LCP. For all three illumination wavelengths, the intensity recorded at half the wavelength is approximately an order of magnitude larger than that of the multiphoton background. This observation indicates that the signal observed is indeed second-harmonic scattering and is not due to other nonlinear optical effects, such as supercontinuum emission or three-photon luminescence. Second-harmonic scattering should also exhibit a square dependence on the incident power, and as a second-harmonic chiroptical effect, a difference for LCP and RCP illumination should be present.

**Figure 3:**
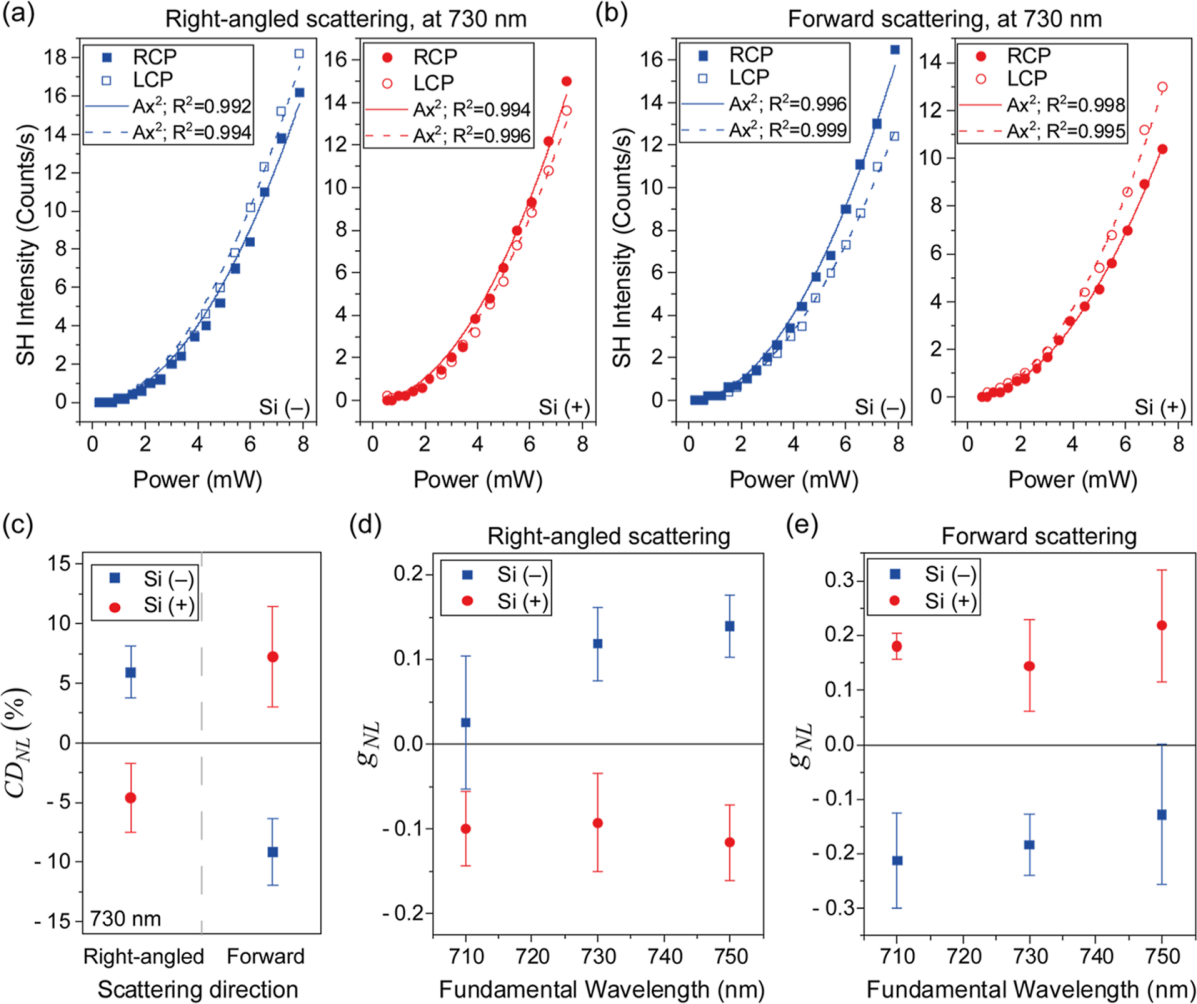
Demonstration of second-harmonic chiroptical Tyndall scattering in Si nanohelices. In (a,b), the second-harmonic (SH) intensity is plotted for RCP and LCP light, respectively, versus incident laser power at a wavelength (λ) of 730 nm. The data points indicate median values of 90 measurements. The data point fits are to the equation *y* = *Ax*^2^ and the *R*^2^ values are provided. The data in parts (a) and (b) correspond to right-angled scattering (with respect to the direction of the incident beam) and to forward scattering, respectively. To clearly illustrate the chiroptical effect, the nonlinear CD corresponding to the data in (a,b) is presented in (c). In (d,e), the mean nonlinear *g*-factor (*g*_NL_) values are presented as a function of the wavelength of illumination, for Si(−) and Si(+). The two chiral forms of Si nanohelices are clearly distinguishable in both right-angled (d) and forward (e) scattering.

The graphs in Figure 3a correspond to the second-harmonic intensity as a function of the incident laser power. The data were obtained for illumination at 730 nm and detection at a right angle to the direction of illumination. The empty and full symbols indicate the polarization state of the incident light (LCP and RCP light, respectively). In each case, the lines are excellent square law fits, with *R*^2^ > 0.99, which is consistent with second-harmonic scattering. Further confirmation is provided in Figure 3b, where the second-harmonic intensity versus incident laser power is plotted for forward scattering. Here again, excellent square law fits are observed for Si(−) and Si(+).

Having demonstrated that the signals detected are indeed due to second-harmonic Tyndall scattering, the chiroptical effect is well-illustrated upon considering the nonlinear CD, as shown in Figure 3c. In this figure, all the data from Figure 3a,b are combined and the nonlinear CD is calculated using
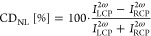
3where *I*_LCP_^2ω^ and *I*_RCP_^2ω^ correspond to the intensity of light detected for LCP and RCP illumination, respectively. Clearly, the CD_NL_ values for Si(−) and Si(+) are well-separated with no overlap between error bars. The effect can also be illustrated using the nonlinear asymmetry *g* factor
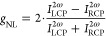
4

Figure 3d,e presents *g*_NL_ versus wavelength from Si(−) and Si(+), in the case of right-angled and forward scattering, respectively. In Figure 3d, despite a slight overlap of the error bars at 710 nm, the Si enantiomorphs are well-resolved, and the *g*_NL_ values are around ±0.1. In Figure 3e, Si(−) and Si(+) are well-resolved, with no overlap of the error bars, and the *g*_NL_ values are in the range ± (0.15 to 0.2). We note that in all of our data, the chiroptical behavior for forward and right-angled scattering is opposite. To understand this difference in sign, we needed to examine the physical origin of the effect.

While it is accepted that Rayleigh scattering occurs for very small particles (dimensions < λ/10), the exact limit of validity for this approximation depends on the refractive index of the particles.^47^ Mie scattering occurs for large particles (dimensions ≥2λ), though here again the exact limit depends on particle shape and on the refractive index.^48^ Between the Rayleigh and Mie ranges, an intermediate particle scattering regime takes place that has been referred to as the “Rayleigh–Mie transition zone”,^49^ and it is characteristic of the Tyndall effect,^50^ especially for suspensions of particles with dimensions in the range ≈λ/10 to ≈2λ/.^51,52^

To capture the essential physical processes at work, we can proceed from the formula for the hyper-Rayleigh scattering intensity, which is given by^53^
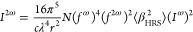
5where β is the hyperpolarizability, ω indicates the frequency, *N* is the concentration of the scatterers, *c* is the speed of light, λ is the wavelength, *f*^ω^ and *f*^2ω^ are the local field factors at the fundamental and second-harmonic frequency, respectively, *I* is the intensity, *r* is the distance to the scattering center, and the bracket indicates the orientational averaging. This formula can be extended toward a regime of larger particles by including a phase relation between different parts of the scatterers—in this case, we first take the sum of the fields and then we square the total to obtain the intensity (similar to the method described in ref (54)). We can also think of the second-harmonic Tyndall scattering process in terms of Fermi’s golden rule. Within this framework, the lower of the two intermediary excited states (near-resonant virtual) is an energy state at the fundamental wavelength that can be probed by linear optical techniques. The higher-level intermediary excited state is at the second-harmonic wavelength—these too can be probed by linear optical techniques. In high-refractive-index dielectrics, lower and upper state transitions can be enhanced by dielectric resonances. Hyper-Tyndall scattering is highly sensitive to such resonances because the local field factors in eq 5 are power laws. The coupling between initial and final states is provided by the frequency-conversion process, i.e., the values of the hyperpolarizability, which is a third-rank tensor. For chiroptical second-harmonic (hyper-)Tyndall scattering, we need to take into account additional nonlinear optical tensors that interact with the hyperpolarizability to produce the effect.

To establish the angular dependence of chiroptical second-harmonic Tyndall scattering, we proceed using fundamental principles.^55^ Three (meta)molecular response tensors are implicated. One is the (meta)molecular hyperpolarizability β, based on electric dipole (E1) interactions with each of the two input frequency photons and with the harmonic frequency output photon. The E1^3^ nature of β gives it an odd spatial parity. The interference of β with tensors of even parity can arise only in systems of broken symmetry and is thereby manifested in chirally responsive interactions. There are four tensors of even parity that dominate such effects. Following previously established nomenclature,^56^ we write these as ^α^*J*, ^β^*J*, ^α^*K*, and ^β^*K* (where the index ^β^ should not be confused with the hyperpolarizability β).

The *J* tensors are of E1^2^M1 character, M1 denoting a transition magnetic dipole; the *K* tensors are E1^2^E2, where E2 signifies a transition electric quadrupole. In the tensor ^α^*J*, a magnetic dipole interaction occurs in the harmonic photon output; in ^β^*J*, this form of interaction is associated with one of the input photons. Equally, ^α^*K* represents harmonic emission from an electric quadrupole interaction, while in ^β^*K*, the quadrupole coupling occurs with an input photon.

**Figure 4:**
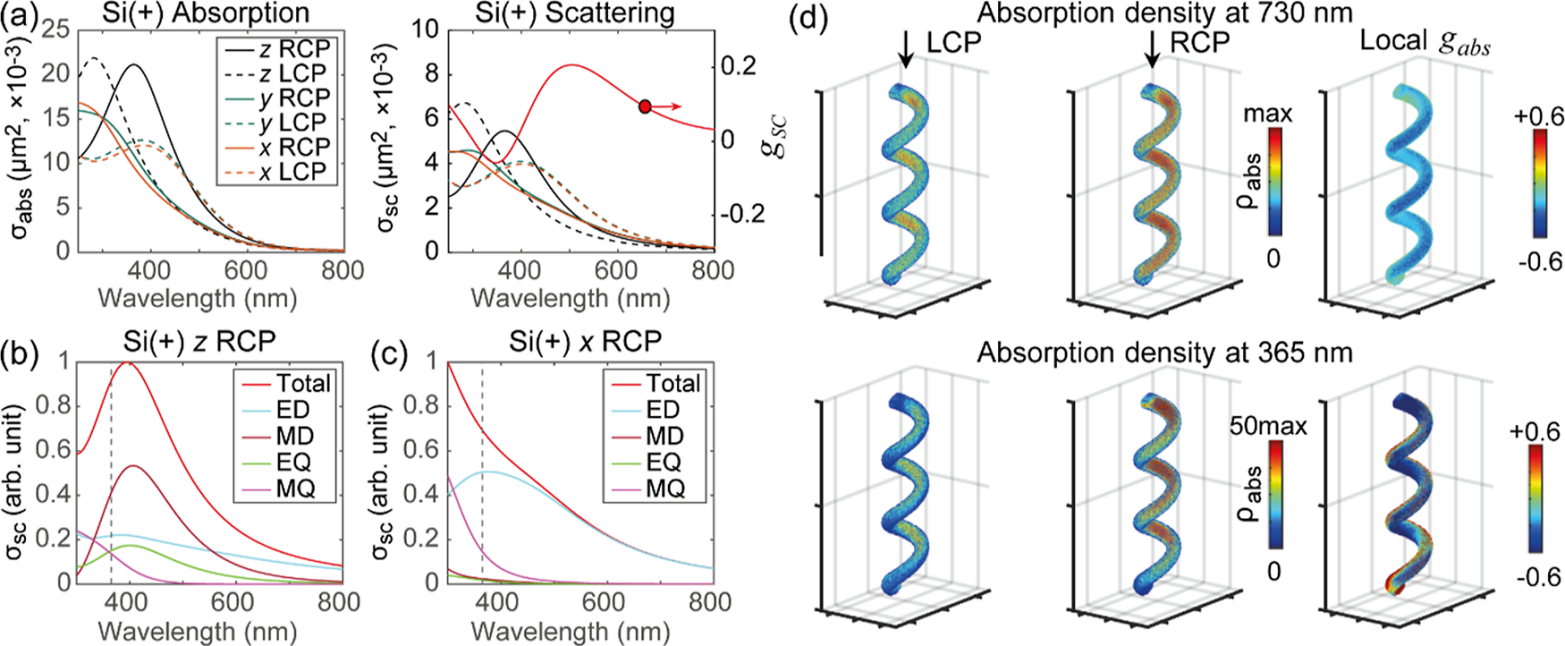
Chirality of the scattered second-harmonic field strongly depends on the nanohelix orientation: in the Si(+) sample, *z*-oriented nanohelices strongly scatter RCP light to the right angle. (a) Absorption (left) and scattering (right) cross-section for Si(+) nanohelices oriented along the three Cartesian directions, illuminated in water, under normal incidence with LCP or RCP light. In each case, the *z*-oriented nanohelices contribute the most. Multipole expansion for the Si(+) nanohelix, excited with RCP light, and oriented in (b) *z*-direction and (c) *x*-direction, shows prevalent contribution of resonant modes in the range of second-harmonic wavelengths; the total scattering is resolved into electric dipole (ED), magnetic dipole (MD), electric quadrupole (EQ), and magnetic quadrupole (MQ). (d) Absorption density in Si(+) helices oriented along the *z*-direction, with LCP and RCP illumination, at 730 nm and at 365 nm. The 3D absorption density ρ_abs_ and local *g*_abs_ distributions are shown.

Figure 1d shows that in our samples, the absorbance is much stronger in the wavelength region of harmonic emission than in that of the input photons. This result is also in agreement with the calculated absorption cross section in Figure 4a. The calculated scattering cross sections in Figure 4b,c illustrate the fact that the region of high absorption can be expected to contain sizable magnetic dipole and electric quadrupole contributions; this is not the case for the wavelength region of the input photons. Therefore, we can make the assumption that ^β^*J* = ^β^*K* = 0.

As can be shown by irreducible tensor methods (see Supporting Information),^56^ the maximum number of rotational invariants that can arise from the β*J* and β*K* products altogether is 13 (six from β^α^*J* and seven from β^α^*K*). In fact, only 8 arise in the explicit rate equations recently rederived by Bonvicini et al.,^57^ and these are given in Table 1, where *k* is the wavenumber of the fundamental frequency pump.

**Table 1:** Linearly Independent (Meta)molecular Invariants Responsible for Chiroptical Second-Harmonic Scattering; the Subscripts λ, μ, ν, ο, π, and ρ Represent the Six Free Cartesian Indices

*j*_1_ = Im β_λλμ_ ^α^*J*_νμν_	*k*_1_ = *k* β_λμμ_ ^α^*K*_οπρρ_ ε_λοπ_	*k*_3_ = *k* β_λμν_ ^α^*K*_οπνμ_ ε_λοπ_	*k*_5_ = *k* β_λμν_ ^α^*K*_νπρρ_ ε_λμπ_
*j*_2_ = Im β_λμν_ ^α^*J*_μλν_	*k*_2_ = *k* β_λμν_ ^α^*K*_ονρρ_ ε_λμο_	*k*_4_ = *k* β_λμν_ ^α^*K*_οππν_ ε_λμο_	*k*_17_ = *k* β_λμν_ ^α^*K*_νπρρ_ ε_λμπ_

This full set subsumes 14 that arise from β*K* products, featured in the results for twisted light recently identified by Forbes.^58^ Each of the (meta)molecular response parameters represents a different sum of products of components of β with components of the *J* and *K* tensors.

The entirety of the general result for a chirality-sensitive difference in the harmonic intensity, emitted at an arbitrary angle with respect to the input beam, is expressible in terms of the *j* and *k* coefficients as follows
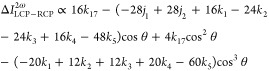
6where θ is defined as cos^–1^(−**k̂**·**k̂**′), with **k̂**, **k̂**′ the unit propagation vectors for the input pump and detected harmonic, respectively. Note that coherent second-harmonic generation in the forward direction is forbidden in any isotropic fluid—as indeed is the coherent generation of any harmonic using circularly polarized light^59^ (except for under the highly intense conditions that produce high-order harmonics).^60^ Hence, the second-harmonic signal detected at any angle, in the cases studied here, can result from only the incoherent form of interaction that generates the above result. The explicit results for forward and right-angled scattering are as follows
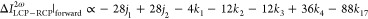
7
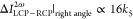
8

The orders of magnitude of each (meta)molecular invariant might be anticipated to be broadly similar, but there is no basis for supposing them to have the same sign. Accordingly, the differently weighted linear combinations in eqs 7 and 8 cannot be expected to display any correlation. In particular, the sign of the chiroptical second-harmonic effect at any angle and at a single wavelength cannot be interpreted as indicating the specific handedness of the scatterer.

To complete the picture from eq 5, we need to consider the local field enhancements at the fundamental and the second-harmonic frequency. These can be illustrated with electromagnetic simulations. Figure 4a thus presents the calculated spectra of the absorption (left) and scattering cross sections (right) along with *g*_sc_ (eq 2). As expected, switching the helix handedness inverts the behavior for the RCP and LCP polarizations (Figure S5). Moreover, the spectral response to LCP and RCP light depends strongly on the orientation of the nanohelices; when the nanohelices are oriented with their long axis along the *z*-direction, there is a strong absorption for RCP light, leading to broad negative absorption CD across the visible range (Figure S6, this result also agrees with ensembles of similar vertical helices in ref (48)). When the helices are oriented perpendicular to the light wave vector, LCP light is more absorbed (Figure S7). Averaging over the three Cartesian directions of nanohelix orientations leads to *g*_SC_ ≈ −0.05 around 365 nm (red line in Figure 4a); when calculated for the *z*-oriented helix only, *g*_SC_ ≈ −0.3 around 365 nm. Far-field scattering distributions at fundamental wavelengths show that, as expected, the chiroptical response in the linear regime cannot be directly correlated with second-harmonic response; in the linear case, there is no inversion of the chiroptical signature between forward and right-angled scattering, Figures S8–S10.

To show the largest local field enhancements, we simulate the *z*-oriented nanohelix excited at 730 and 365 nm, with LCP and RCP light, Figure 4d. The absorption density (ρ_abs_) distribution and local *g*_abs_ are shown, where local *g*_abs_ = 2·[ρ_abs_^LCP^ – ρ_abs_^RCP^]/[ρ_abs_^LCP^ + ρ_abs_^RCP^]. The local *g*_abs_ value mostly varies from −0.2 to −0.5.

## Conclusions

In summary, we report the observation of chiroptical second-harmonic (hyper-)Tyndall scattering in high refractive dielectrics (specifically in Si) and present a theoretical analysis that reveals its origin. Due to their pronounced displacement current resonances, with significant multipolar contributions, high-refractive-index dielectrics (especially Si) are of prime interest for developing optical applications, for instance, in metasurfaces. To avoid any confusion with supercontinuum emission or multiphoton luminescence, we show that the second-harmonic signal is several times stronger than the multiphoton background. We then demonstrate that the observed signal intensity versus incident light power fits to a square law, with *R*^2^ > 0.99 in each case. We show that the second-harmonic intensity depends on the handedness of circularly polarized light and on the chirality of the scatterers. This behavior is demonstrated at three separate wavelengths, for both forward and right-angled (with respect to the direction of incident light) scattering.

We present a theoretical analysis that pinpoints the origin of the observed chiroptical effect down to the (meta)molecular invariants within the irreducible tensors that describe the interaction between electric dipoles and transition electric quadrupoles and magnetic dipoles. While the general theory includes up to 31 rotational invariants (see General Theory in the Supporting Information), in the case of Si nanohelices, the results from numerical simulations allow us to reduce this number to 13, with only 8 contributing to forward scattering and a single contribution to the right-angled scattering. We also discuss the importance of local field factors and the relationship between the chiroptical effects in the second-harmonic and linear optical regime.

Our work demonstrates that it is possible to characterize the chirality of high-refractive-index dielectric nanoparticles in a completely isotropic environment (in liquids, free of all of the artifacts associated with ordering nanoparticle arrays) based on their nonlinear and pronounced multipolar properties. It also confirms that CHS is a general method that is not solely restricted to plasmonic and other semiconducting nanomaterials. To observe further enhanced CHS, it would be interesting to combine materials with different properties. Such hybrid materials^61^ could selectively combine plasmonic, excitonic, and dielectric resonances at wavelengths of choice and with strong, nanoengineered multipolar responses. To fully benefit from the wavelength tunability of such resonances in nanostructured materials, it will be important to develop second-harmonic chiroptical scattering spectra over a larger wavelength range. The theoretical methods presented here can be expanded and adapted to other forms of CHS, including in materials where the effect has not yet been observed, such as nanocrystals, quantum dots, organic–inorganic compounds, polymers, etc.

## Methods

### Fabrication of the Si Nanohelices

Low p-type doped (100) oriented single-crystal Si substrates (from University Wafers Inc.) were utilized as the main substrate. To disperse the helical nanostructures in a liquid environment, a “sacrifice” thin film layer on the substrate is necessary. Uniform and conformal ZnO ultrathin films on top of the Si substrate (at a steady temperature of 250 °C and pressure of 0.2 Torr) were fabricated using the oxygen plasma-enhanced atomic layer deposition (ALD) technique (Fiji F200 Veeco CNT). Prior to the ALD of the ZnO process, a predeposition oxygen plasma (300 W) was employed to remove the impurities and contaminants from the surface. The reactant and coreactant precursors were dimethylzinc {Zn[(CH_3_)_2_]} and oxygen plasma, respectively. Argon was employed as the purging gas source. Based on dynamic dual box model analysis of in situ spectroscopic ellipsometry data, an approximately 7 nm-thick ZnO ultrathin film was measured on the surface.^62^

A custom-built, ultrahigh vacuum glancing angle deposition (GLAD) system was utilized in the fabrication of helical scatterers. The base pressure of the deposition system was measured at 1.0 × 10^–8^ mbar, prior to the fabrication of the nanostructures. An electron beam evaporation technique was utilized in GLAD, and the particle flux impinged on the substrate surface with an oblique angle (θ_flux_) of 85° from the surface normal. Due to the integration of a quartz crystal microbalance deposition controller with the deposition chamber, the real-time thickness monitoring enabled the control of the deposition rate during the growth of helices, and the deposition rate was maintained at 1.34 Å/s. The sample manipulation arm of GLAD plays a key role in the creation of helical morphology from the incoming particle flux on the sample substrate. Hence, the sample stage was monotonously rotated with a speed of 0.9 deg/s.

To disperse the helical nanostructures, a piece of the sample was placed in a vial of filtered water and then sonicated for 30 min. A small amount of solution was extracted by a micropipet and deposited on a lacey carbon grid. The scanning\transmission electron microscopy (STEM) work was performed with an FEI Tecnai Osiris at 200 kV. Thus, we performed bright-field imaging in TEM and high-angular annular dark-field imaging in STEM and used a ChemiSTEM for energy-dispersive X-ray spectroscopy in STEM. The high-resolution SEM images were obtained with a Helios NanoLab 660. The SEM images were obtained as the field emission, and the beam current parameters were chosen as 5 kV and 0.1 nA.

### Sample Preparation for Nonlinear Optical Characterization

To prepare Si(−) suspensions, a 1 cm × 1 cm wafer was submerged in 2 mL of distilled water and sonicated for 5 min. It was then left to stand for 1 min and sonicated again for a another minute. 1 mL of the suspension was pipetted into a cuvette for nonlinear measurements. Si(+) suspensions were prepared by adjusting the wafer surface/water volute ratio to keep the concentration similar to that of the Si(−) samples.

### Linear Optical Characterization

Solutions of Si nanohelices in fused quartz cuvettes were characterized in a Jasco J-810 CD spectrometer in the spectral region of 220–800 nm. The measured CD spectrum of the reference sample (a cuvette with water) was subtracted from the spectra of the investigated samples. The path length in the cuvette was 10 mm. The time-per-point was set to 2 s and the data pitch to 1 nm. Each spectrum was measured three times before taking an average. The bandwidth was set to 2 nm, and the scanning speed was set to 50 nm/min.

### Nonlinear Optical Characterization

A Ti/sapphire laser (repetition rate of 80 MHz; pulse width of 75 fs) was the source for the nonlinear experiments. An optical chopper with a 3.3% duty cycle modulated the laser beam at a frequency of 41 Hz. An achromatic half-wave plate designed to work in the region of the 400–800 nm spectral range was placed before an uncoated Glan-Laser calcite polarizer for power control. After passing through the polarizer, an achromatic quarter-wave plate (design wavelength range 690–1200 nm) controlled the polarization state of the excitation beam. LCP light is defined from the point of view of the source, looking along the direction of propagation such that the electric field of light traces a helix in space that rotates to the left. An antireflection-coated achromatic doublet lens (focal length 30 mm) focused the beam into a quartz cuvette filled with 1 mL of solution containing nanohelices. A pair of long-pass filters placed before the focusing lens (cutoff wavelength 665 nm) was used to remove any residual second-harmonic light. In experiments measuring scattering at a right angle, an antireflection-coated achromatic doublet lens (focal length 25.4 mm) was used to collimate scattered light. A bandpass filter (transmission range 335–610 nm) and another antireflection-coated lens (focal length 100 mm) focused the collected light onto the photocathode of a PMT. In experiments performed in transmission geometry, an antireflection-coated 25.4 mm focal-length lens was placed after the cuvette and was followed by a colored glass bandpass filter (also with a 355–610 nm transmission window). In all experiments, hard-coated bandpass filters with a 10 nm full-width at half-maximum of their transmission peak were placed in front of the detector to isolate scattered light within the desired wavelength range. The signal from the PMT was preamplified five times before entering a photon counter. The photon counter was used in the gated regime. The results presented in Figure 3 were obtained with 7 mW incident laser power.

### Numerical Simulations

Full-wave electromagnetic 3D simulations were performed by using the FDTD module in Lumerical. We first simulated a dense ensemble of vertically standing nanohelices on a substrate in the air, which corresponds to the sample before the solution preparation. As nanohelices with such dimensions were previously experimentally characterized,^44^ we ensured that the simulated differential absorption of such Si(+) and Si(−) metamaterials on a substrate provided good agreement with the measurements and simulations of the previous work. Next, scattering and absorption properties of a single nanohelix were simulated in water (FDTD medium with a refractive index of 1.33). The nanohelix was surrounded by a simulation region defined by perfectly matched layers (PMLs) in all directions. The initial simulations included broadband excitation in the 200–2000 nm range; the distance between the nanohelix and PMLs was therefore set to more than one maximum wavelength to prevent simulation instabilities. The orientation of the nanohelix was set to *x*-, *y*-, or *z*-direction and surrounded by a localized refinement region in all directions, while the overall mesh accuracy was set to 4. Dispersive dielectric constants of polycrystalline Si were taken from the experimental data. The nanohelix was excited by a circularly polarized source from the top (the beam was traveling in the negative *z*-direction). All simulations were performed at normal angle of incidence, as in the experiment. To obtain the circular polarization, two total-field-scattered-field (TFSF) sources were combined; they were perpendicular to each other and had a phase offset of +90° or −90°, while having all of the other properties equal. In this work, +90° (−90°) corresponds to RCP (LCP) excitation. The TFSF source divides the FDTD region into the one inside TF (total field) and the other outside SF (scattered field). Therefore, the absorption and scattering cross sections were calculated by surrounding the nanohelix with a box of 6 power monitors in the total and in the scattered field, respectively. Next, the absorption distribution was calculated at single wavelengths by monitoring the absorption density across the nanohelix volume. The scattered far-field distribution was calculated by projecting the fields measured by power monitors in the scattered region. As the source is exciting the nanohelix in the negative *z*-direction, this direction corresponds to the forward scattering in the experiment, while *x*-direction corresponds to the right-angled scattering. To resolve contributions of different modes to the total scattering cross section of the single nanohelix, we further used MENP, an open-source MATLAB-based solver for multipole expansion,^63^ in combination with four-dimensional complex electromagnetic field extraction from Lumerical.

## Data Availability

The data that support the findings of this study are openly available in the repository of the University of Bath at 10.15125/BATH-01364.

## Supporting Information Available

Dielectric function of the Si nanohelices, section on sample preparation, section on power dependence measurements, section on general theory, and section on numerical simulations (PDF)

## References

[ref1] Lu J.; Xue Y.; Kotov N. A. Emerging Trends in Chiral Inorganic Nanostructures. Isr. J. Chem. 2021, 61, 851–862. 10.1002/ijch.202100076.

[ref2] Ma W.; Xu L.; Wang L.; Xu C.; Kuang H. Chirality-Based Biosensors. Adv. Funct. Mater. 2019, 29, 1805512 10.1002/adfm.201805512.

[ref3] Yamanishi J.; Ahn H.-Y.; Yamane H.; Hashiyada S.; Ishihara H.; Nam K. T.; Okamoto H. Optical gradient force on chiral particles. Sci. Adv. 2022, 8, eabq2604 10.1126/sciadv.abq2604. 36129977 PMC9491721

[ref4] Schamel D.; Pfeifer M.; Gibbs J. G.; Miksch B.; Mark A. G.; Fischer P. Chiral Colloidal Molecules And Observation of The Propeller Effect. J. Am. Chem. Soc. 2013, 135, 12353–12359. 10.1021/ja405705x. 23883328 PMC3856768

[ref5] Wu X.; Hao C.; Kumar J.; Kuang H.; Kotov N. A.; Liz-Marzán L. M.; Xu C. Environmentally responsive plasmonic nanoassemblies for biosensing. Chem. Soc. Rev. 2018, 47, 4677–4696. 10.1039/C7CS00894E. 29737984

[ref6] Li S.; Xu L.; Sun M.; Wu X.; Liu L.; Kuang H.; Xu C. Hybrid Nanoparticle Pyramids for Intracellular Dual MicroRNAs Biosensing and Bioimaging. Adv. Mater. 2017, 29, 1606086 10.1002/adma.201606086. 28221715

[ref7] Xu L.; Wang X.; Wang W.; Sun M.; Choi W. J.; Kim J.-Y.; Hao C.; Li S.; Qu A.; Lu M.; Wu X.; Colombari F. M.; Gomes W. R.; Blanco A. L.; de Moura A. F.; Guo X.; Kuang H.; Kotov N. A.; Xu C. Enantiomer-dependent immunological response to chiral nanoparticles. Nature 2022, 601, 366–373. 10.1038/s41586-021-04243-2. 35046606

[ref8] Sun M.; Xu L.; Qu A.; Zhao P.; Hao T.; Ma W.; Hao C.; Wen X.; Colombari F. M.; de Moura A. F.; Kotov N. A.; Xu C.; Kuang H. Site-selective photoinduced cleavage and profiling of DNA by chiral semiconductor nanoparticles. Nat. Chem. 2018, 10, 821–830. 10.1038/s41557-018-0083-y. 30030537

[ref9] Gao R.; Xu L.; Sun M.; Xu M.; Hao C.; Guo X.; Colombari F. M.; Zheng X.; Král P.; de Moura A. F.; Xu C.; Yang J.; Kotov N. A.; Kuang H. Site-selective proteolytic cleavage of plant viruses by photoactive chiral nanoparticles. Nat. Catal. 2022, 5, 694–707. 10.1038/s41929-022-00823-1.

[ref10] Arad E.; Bhunia S. K.; Jopp J.; Kolusheva S.; Rapaport H.; Jelinek R. Lysine-Derived Carbon Dots for Chiral Inhibition of Prion Peptide Fibril Assembly. Adv. Ther. 2018, 1, 1800006 10.1002/adtp.201800006.

[ref11] Jiang S.; Chekini M.; Qu Z. B.; Wang Y.; Yeltik A.; Liu Y.; Kotlyar A.; Zhang T.; Li B.; Demir H. V.; Kotov N. A. Chiral Ceramic Nanoparticles and Peptide catalysis. J. Am. Chem. Soc. 2017, 139, 13701–13712. 10.1021/jacs.7b01445. 28803469

[ref12] Hao C.; Gao R.; Li Y.; Xu L.; Sun M.; Xu C.; Kuang H. Chiral Semiconductor Nanoparticles for Protein Catalysis and Profiling. Angew. Chem., Int. Ed. 2019, 58, 7371–7374. 10.1002/anie.201902673. 30950141

[ref13] Lee H. E.; Ahn H. Y.; Mun J.; Lee Y. Y.; Kim M.; Cho N. H.; Chang K.; Kim W. S.; Rho J.; Nam K. T. Amino-acid- and peptide-directed synthesis of chiral plasmonic gold nanoparticles. Nature 2018, 556, 360–365. 10.1038/s41586-018-0034-1. 29670265

[ref14] Cheng J.; Hao J.; Liu H.; Li J.; Li J.; Zhu X.; Lin X.; Wang K.; He T. Optically Active CdSe-Dot/CdS-Rod Nanocrystals with Induced Chirality and Circularly Polarized Luminescence. ACS Nano 2018, 12, 5341–5350. 10.1021/acsnano.8b00112. 29791135

[ref15] Collins J. T.; Rusimova K. R.; Hooper D. C.; Jeong H.-H.; Ohnoutek L.; Pradaux-Caggiano F.; Verbiest T.; Carbery D.; Fischer P.; Valev V. K. First Observation of Optical Activity in Hyper-Rayleigh Scattering. Phys. Rev. X 2019, 9, 011024 10.1103/PhysRevX.9.011024.

[ref16] Ohnoutek L.; Kim J.-Y.; Lu J.; Olohan B. J.; Răsădean D. M.; Dan Pantoş G.; Kotov N. A.; Valev V. K. Third-harmonic Mie scattering from semiconductor nanohelices. Nat. Photonics 2022, 16, 126–133. 10.1038/s41566-021-00916-6.

[ref17] Ohnoutek L.; Cho N. H.; Murphy A. W. A.; Kim H.; Răsădean D. M.; Pantoş G. D.; Nam K. T.; Valev V. K. Single Nanoparticle Chiroptics in a Liquid: Optical Activity in Hyper-Rayleigh Scattering from Au Helicoids. Nano Lett. 2020, 8, 5792–5798. 10.1021/acs.nanolett.0c01659. PMC7467767 32579377

[ref18] Ohnoutek L.; Olohan B. J.; Jones R. R.; Zheng X.; Jeong H.-H.; Valev V. K. Second-harmonic Rayleigh scattering optical activity of single Ag nanohelices in a liquid. Nanoscale 2022, 14, 3888–3898. 10.1039/D1NR06800H. 35212336

[ref19] Ohnoutek L.; Jeong H.-H.; Jones R. R.; Sachs J.; Olohan B. J.; Răsădean D. M.; Pantoş G. D.; Andrews D. L.; Fischer P.; Valev V. K. Optical Activity in Third-Harmonic Rayleigh Scattering: A New Route for Measuring Chirality. Laser Photonics Rev. 2021, 15, 2100235 10.1002/lpor.202100235.

[ref20] Shen Y. R. The Principles of Nonlinear Optics; John Wiley & Sons: New York, 1984; p 479.

[ref21] Boyd R. W. Nonlinear Optics, 3rd ed.; Academic Press, Inc., 2008; p 122.

[ref22] Koshelev K.; Tonkaev P.; Kivshar Y. Nonlinear chiral metaphotonics: a perspective. Adv. Photonics 2023, 5, 064001 10.1117/1.AP.5.6.064001.

[ref23] Valev V. K.; Kirilyuk A.; Dalla Longa F.; Kohlhepp J. T.; Koopmans B.; Rasing T. Observation of periodic oscillations in magnetization-induced second harmonic generation at the Mn/Co interface. Phys. Rev. B: Condens. Matter Mater. Phys. 2007, 75, 012401 10.1103/PhysRevB.75.012401.

[ref24] Kivshar Y. The Rise of Mie-tronics. Nano Lett. 2022, 22, 3513–3515. 10.1021/acs.nanolett.2c00548. 35446566

[ref25] Yavas O.; Svedendahl M.; Dobosz P.; Sanz V.; Quidant R. On-a-chip Biosensing Based on All-Dielectric Nanoresonators. Nano Lett. 2017, 17, 4421–4426. 10.1021/acs.nanolett.7b01518. 28616986

[ref26] Bontempi N.; Chong K. E.; Orton H. W.; Staude I.; Choi D.-Y.; Alessandri I.; Kivshar Y.; Neshev D. N. Highly sensitive biosensors based on all-dielectric nanoresonators. Nanoscale 2017, 9, 4972–4980. 10.1039/C6NR07904K. 28382350

[ref27] Zograf G. P.; Ryabov D.; Rutckaia V.; Voroshilov P.; Tonkaev P.; Permyakov D. V.; Kivshar Y. S.; Makarov S. V. Stimulated Raman Scattering from Mie-Resonant Subwavelength Nanoparticles. Nano Lett. 2020, 20, 5786–5791. 10.1021/acs.nanolett.0c01646. 32579376

[ref28] Reineke B.; Sain B.; Zhao R.; Carletti L.; Liu B.; Huang L.; De Angelis C.; Zentgraf T. Silicon Metasurfaces for Third Harmonic Geometric Phase Manipulation and Multiplexed Holography. Nano Lett. 2019, 19, 6585–6591. 10.1021/acs.nanolett.9b02844. 31405278 PMC6746059

[ref29] Hsieh P.-Y.; Fang S. L.; Lin Y.-S.; Huang W. H.; Shieh J.-M.; Yu P.; Chang Y.-C. Integrated metasurfaces on silicon photonics for emission shaping and holographic projection. Nanophotonics 2022, 11, 4687–4695. 10.1515/nanoph-2022-0344. PMC11501560 39634746

[ref30] Liang X.; Yu Y.; Xu X.; Fu Y. H.; Valuckas V.; Paniagua-Dominguez R.; Kuznetsov A. I. Near unity transmission and full phase control with asymmetric Huygens’ dielectric metasurfaces for holographic projection. Appl. Opt. 2022, 61, B165–B170. 10.1364/AO.444728. 35201137

[ref31] Wei Q.; Sain B.; Wang Y.; Reineke B.; Li X.; Huang L.; Zentgraf T. Simultaneous Spectral and Spatial Modulation for Color Printing and Holography Using All-Dielectric Metasurfaces. Nano Lett. 2019, 19, 8964–8971. 10.1021/acs.nanolett.9b03957. 31693377 PMC6910142

[ref32] Zhou H.; Sain B.; Wang Y.; Schlickriede C.; Zhao R.; Zhang X.; Wei Q.; Li X.; Huang L.; Zentgraf T. Polarization-Encrypted Orbital Angular Momentum Multiplexed Metasurface Holography. ACS Nano 2020, 14, 5553–5559. 10.1021/acsnano.9b09814. 32348122 PMC7254835

[ref33] Fenollosa R.; Garcia-Rico E.; Alvarez S.; Alvarez R.; Yu X.; Rodriguez I.; Carregal-Romero S.; Villanueva C.; Garcia-Algar M.; Rivera-Gil P.; de Lera A. R.; Parak W. J.; Meseguer F.; Alvarez-Puebla R. A. Silicon particles as trojan horses for potential cancer therapy. J. Nanobiotechnol. 2014, 12, 35 10.1186/s12951-014-0035-7. PMC4428529 25223512

[ref34] Valuckas V.; Paniagua-Dominguez R.; Maimaiti A.; Patra P. P.; Wong S. K.; Verre R.; Käll M.; Kuznetsov A. I. Fabrication of Monodisperse Colloids of Resonant Spherical Silicon Nanoparticles: Applications in Optical Trapping and Printing. ACS Photonics 2019, 6, 2141–2148. 10.1021/acsphotonics.9b00722.

[ref35] Tanaka K.; Arslan D.; Fasold S.; Steinert M.; Sautter J.; Falkner M.; Pertsch T.; Decker M.; Staude I. Chiral Bilayer All-Dielectric Metasurfaces. ACS Nano 2020, 14, 15926–15935. 10.1021/acsnano.0c07295. 33179909

[ref36] Solomon M. L.; Hu J.; Lawrence M.; García-Etxarri A.; Dionne J. A. Enantiospecific Optical Enhancement of Chiral Sensing and Separation with Dielectric Metasurfaces. ACS Photonics 2019, 6, 43–49. 10.1021/acsphotonics.8b01365.

[ref37] Shcherbakov M. R.; Neshev D. N.; Hopkins B.; Shorokhov A. S.; Staude I.; Melik-Gaykazyan E. V.; Decker M.; Ezhov A. A.; Miroshnichenko A. E.; Brener I.; Fedyanin A. A.; Kivshar Y. S. Enhanced Third-Harmonic Generation in Silicon Nanoparticles Driven by Magnetic Response. Nano Lett. 2014, 14, 6488–6492. 10.1021/nl503029j. 25322350

[ref38] Koshelev K.; Tang Y.; Hu Z.; Kravchenko I. I.; Li G.; Kivshar Y. Resonant Chiral Effects in Nonlinear Dielectric Metasurfaces. ACS Photonics 2023, 10, 298–306. 10.1021/acsphotonics.2c01926.

[ref39] Bar-David J.; Levy U. Nonlinear Diffraction in Asymmetric Dielectric Metasurfaces. Nano Lett. 2019, 19, 1044–1051. 10.1021/acs.nanolett.8b04342. 30608703

[ref40] Makarov S. V.; Petrov M. I.; Zywietz U.; Milichko V.; Zuev D.; Lopanitsyna N.; Kuksin A.; Mukhin I.; Zograf G.; Ubyivovk E.; Smirnova D. A.; Starikov S.; Chichkov B. N.; Kivshar Y. S. Efficient Second-Harmonic Generation in Nanocrystalline Silicon Nanoparticles. Nano Lett. 2017, 17 (5), 3047–3053. 10.1021/acs.nanolett.7b00392. 28409641

[ref41] Tyndall J. On the Blue Colour of the Sky, and the Polarization of Light. Proc. Roy. Soc. Lond. 1869, 17, 223.

[ref42] Tyndall J. Six Lectures on Light; Longmans, Green, and Co.: London, 1873; p 155.

[ref43] Khan S. A.; Khan N. Z.; Xie Y.; Abbas M. T.; Rauf M.; Mehmood I.; Runowski M.; Agathopoulos S.; Zhu J. Optical Sensing by Metamaterials and Metasurfaces: From Physics to Biomolecule Detection. Adv. Opt. Mater. 2022, 10, 2200500 10.1002/adom.202200500.

[ref44] Bashir S.; Shamsi A.; Ahmad F.; Hassan M. I.; Kamal M. A.; Islam A. Biophysical Elucidation of Fibrillation Inhibition by Sugar Osmolytes in α-Lactalbumin: Multispectroscopic and Molecular Docking Approaches. ACS Omega 2020, 5, 26871–26882. 10.1021/acsomega.0c04062. 33111013 PMC7581248

[ref45] Gao L.; Ma C.; Wei S.; Kuklin A. V.; Zhang H.; Ågren H. Applications of Few-Layer Nb2C MXene: Narrow-Band Photodetectors and Femtosecond Mode-Locked Fiber Lasers. ACS Nano 2021, 15, 954–965. 10.1021/acsnano.0c07608. 33480253

[ref46] Schmidt D.; Schubert M. Anisotropic Bruggeman effective medium approaches for slanted columnar thin films. J. Appl. Phys. 2013, 114, 083510 10.1063/1.4819240.

[ref47] Bohren C. F.; Huffman D. R. Absorption and Scattering of Light by Small Particles; Wiley, 1998; p 136.

[ref48] van de Hulst H. C. Light Scattering by Small Particles; Dover Publications, 1981; p 445.

[ref49] Mishchenko M. I.; Hovenier J. W.; Travis L. D. Light Scattering by Nonspherical Particles: Theory, Measurements, and Applications; Academic Press, 2000; p 395.

[ref50] Young A. T. Rayleigh scattering. Phys. Today 1982, 35, 42–48. 10.1063/1.2890003.

[ref51] Sharma A. K.; Yang J.-M.; Pandey S.; Wu H.-F. Introducing Tb4+ in (Ce0.09/Eu0.96)Tb0.92Mo1.1O6.93 Metal Oxide at Room Temperature and Its Use in Amyloid Defibrillation. ACS Appl. Mater. Interfaces 2021, 13, 18184–18193. 10.1021/acsami.0c17806. 33826292

[ref52] Hu W.; Ding L.; Cao J.; Liu L.; Wei Y.; Fang Y. Protein Binding-Induced Surfactant Aggregation Variation: A New Strategy of Developing Fluorescent Aqueous Sensor for Proteins. ACS Appl. Mater. Interfaces 2015, 7, 4728–4736. 10.1021/am508421n. 25664917

[ref53] Clays K.; Persoons A.; De Maeyer L. Hyper-Rayleigh scattering in solution. Adv. Chem. Phys. 1994, 85, 455.

[ref54] Verbiest T.; Clays K.; Rodriguez V. Second-harmonic Nonlinear Optical Characterization Techniques: An Introduction; CRC Press, 2009; p 30.

[ref55] Andrews D. L. Rayleigh and Raman optical activity: An analysis of the dependence on scattering angle. J. Chem. Phys. 1980, 72, 4141–4144. 10.1063/1.439643.

[ref56] Andrews D. L. Symmetry-based identification and enumeration of independent tensor properties in nonlinear and chiral optics. J. Chem. Phys. 2023, 158, 034101 10.1063/5.0129636. 36681645

[ref57] Bonvicini A.; Forbes K. A.; Andrews D. L.; Champagne B. Hyper-Rayleigh scattering optical activity: Theory, symmetry considerations, and quantum chemistry applications. J. Chem. Phys. 2023, 158, 204103 10.1063/5.0152784. 37212401

[ref58] Forbes K. A. N onlinear chiral molecular photonics using twisted light: hyper-Rayleigh and hyper-Raman optical activity. J. Opt. 2020, 22, 095401 10.1088/2040-8986/aba0fd.

[ref59] Andrews D. L. Harmonic generation in free molecules. J. Phys. B: At., Mol. Opt. Phys. 1980, 13, 4091–4099. 10.1088/0022-3700/13/20/021.

[ref60] Alon O. E.; Averbukh V.; Moiseyev N. Selection Rules for High Harmonic Generation Spectra. Phys. Rev. Lett. 1998, 80, 3743–3346. 10.1103/PhysRevLett.80.3743.

[ref61] Kilic U.; Hilfiker M.; Ruder A.; Feder R.; Schubert E.; Schubert M.; Argyropoulos C. Broadband Enhanced Chirality with Tunable Response in Hybrid Plasmonic Helical Metamaterials. Adv. Funct. Mater. 2021, 31, 2010329 10.1002/adfm.202010329.

[ref62] Kilic U.; Mock A.; Sekora D.; Gilbert S.; Valloppilly S.; Melendez G.; Ianno N.; Langell M.; Schubert E.; Schubert M. Precursor-surface interactions revealed during plasma-enhanced atomic layer deposition of metal oxide thin films by in-situ spectroscopic ellipsometry. Sci. Rep. 2020, 10, 10392 10.1038/s41598-020-66409-8. 32587273 PMC7316976

[ref63] Hinamoto T.; Fujii M. MENP: An Open-Source MATLAB Implementation of Multipole Expansion for Nanophotonics. OSA Continuum 2021, 4, 1640–1648. 10.1364/OSAC.425189.

