# Supplementary material for: Chiroptical Second-Harmonic Tyndall Scattering from Silicon Nanohelices
Source: ACS Nano. 2024 Jun 17;18(26):16766–75. doi: 10.1021/acsnano.4c02006 (PMC11223486; doi:10.1021/acsnano.4c02006)
Supplement: Supplementary file 1 — nn4c02006_si_001.pdf [file nn4c02006_si_001.pdf]

Chiroptical Second-Harmonic Tyndall Scattering from Silicon Nanohelices

Ben J. Olohan,[1,2] *Emilija Petronijevic,*[3*] *Ufuk Kilic,*[4] *Shawn Wimer,*[4] *Matthew Hilfiker,*[4] *Mathias Schubert,*[4,5] *Christos Argyropoulos,*[6] *Eva Schubert,*[4] *Samuel R. Clowes,*[7] *G. Dan Pantoş,*[7] *David L. Andrews,*[8] *Ventsislav K. Valev*[1,2*]

1. Centre of Photonics and Photonic Materials, University of Bath, Bath, BA2 7AY, UK
2. Centre of Nanoscience and Nanotechnology, University of Bath, Bath, BA2 7AY, UK
3. SBAI Department, La Sapienza University of Rome, Rome, 00161, Italy
4. Department of Electrical and Computer Engineering, University of Nebraska-Lincoln, Lincoln, NE 68588, USA
5. Solid State Physics and NanoLund, Lund University, Box 118, Lund, 22100 Skane, Sweden
6. Department of Electrical Engineering, The Pennsylvania State University, University Park, PA 16803, USA
7. Department of Chemistry, University of Bath, Bath, BA2 7AY, UK.
8. Centre for Photonics and Quantum Science, University of East Anglia, Norwich, NR4 7TJ, UK

Email: emilija.petronijevic@uniroma1.it; v.k.valev@bath.ac.uk

1. Our (almost) dielectric Si nanohelices

The Si nanohelices in our study are almost dielectric. Strictly speaking, if we consider the band gap of Si, both our fundamental and our second-harmonic frequencies are above it, therefore the nanohelices can be said to be semiconducting. On the other hand, if we consider the very low loss tangent of Si in the visible, it would be considered a dielectric.

In the context of light scattering, the term "high refractive index dielectrics" usually emphasizes the ability to access electric and magnetic Mie resonances, to avoid plasmonic loss and to manipulate the directionality of light emission.[1,2] There is emphasis on the near infrared region,[3] but features can be observed in the visible region as well.[4,5,6] In the literature, the terms "low loss dielectrics" and "high refractive index dielectrics" are often used, especially in the field of dielectric metamaterials and metasurfaces.

Our Si nanohelices are not strictly speaking "dielectrics" in the region that we study, based on the bandgap argument. However, in the visible and especially around 730 nm, they appear to have very low dissipation losses (see Figure S1) and are characterized by a low loss tangent, which is typical of dielectrics.

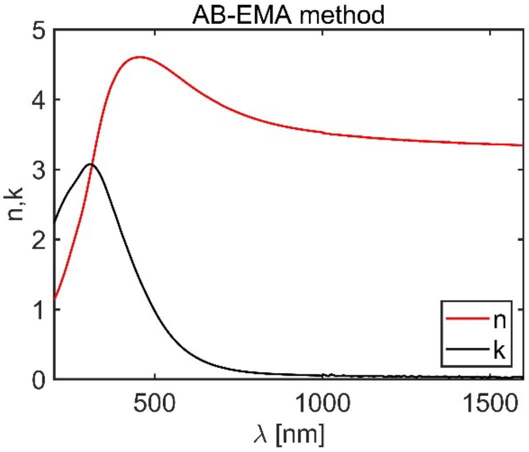

Figure S1: *The dielectric function of Si nanostructures was extracted from Anisotropic Bruggeman Effective medium approach-based, Mueller Matrix Spectroscopic ellipsometry data analysis.*

2. Sample preparation

The Si helices were manufactured on a Si substrate with a thin ZnO layer. The wafers were divided into pieces and sonicated to disperse the helices in distilled water. Figure S2 shows the dimensions of the cut wafer for the Si (-) (a) and Si (+) (b) helices. The wafers are surrounded by a yellow box as a guide for the eye.

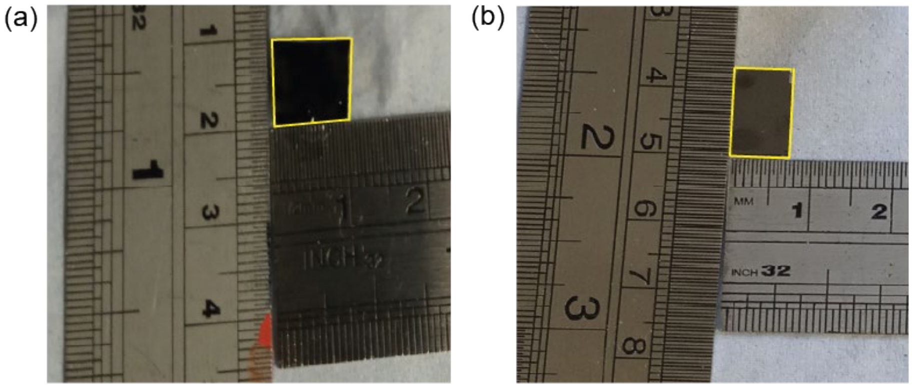

Figure S2: The pieces of the wafers used to prepare the Si (-) (a) and Si (+) (b) samples in solution. Both pieces are surrounded by a yellow box to aid in estimating their size. Both pieces are of similar size and approximately rectangular. Differences in the total area (and hence number of helices) could be corrected by changing the volume of water they were dispersed in.

3. Power dependence Measurements

Power dependence measurements for the Si (-) and Si (+) helices were performed in both right angle and forward scattering for fundamental wavelengths of 710 nm (Figure S3) and 750 nm (Figure S4). Here we measure second harmonic wavelengths of 355 nm and 375 nm respectively. Power dependence for 730 nm can be found in Figure 3 in the main manuscript. In both cases, the data fit excellently with a fit of $y=Ax^2$, showing good agreement with a second order nonlinear effect. Additionally, there are clear signs of chirality in Figure S3 (b) and Figure S4 (a) and (b), that are consistent with Figure 3 in the main manuscript. We additionally show the nonlinear CD (Cd^{NL}_{ext}) for scattering in right angle and forward directions for both enantiomorphs, in figure S3 (c) and figure S4 (c)for 710 and 750 nm respectively.

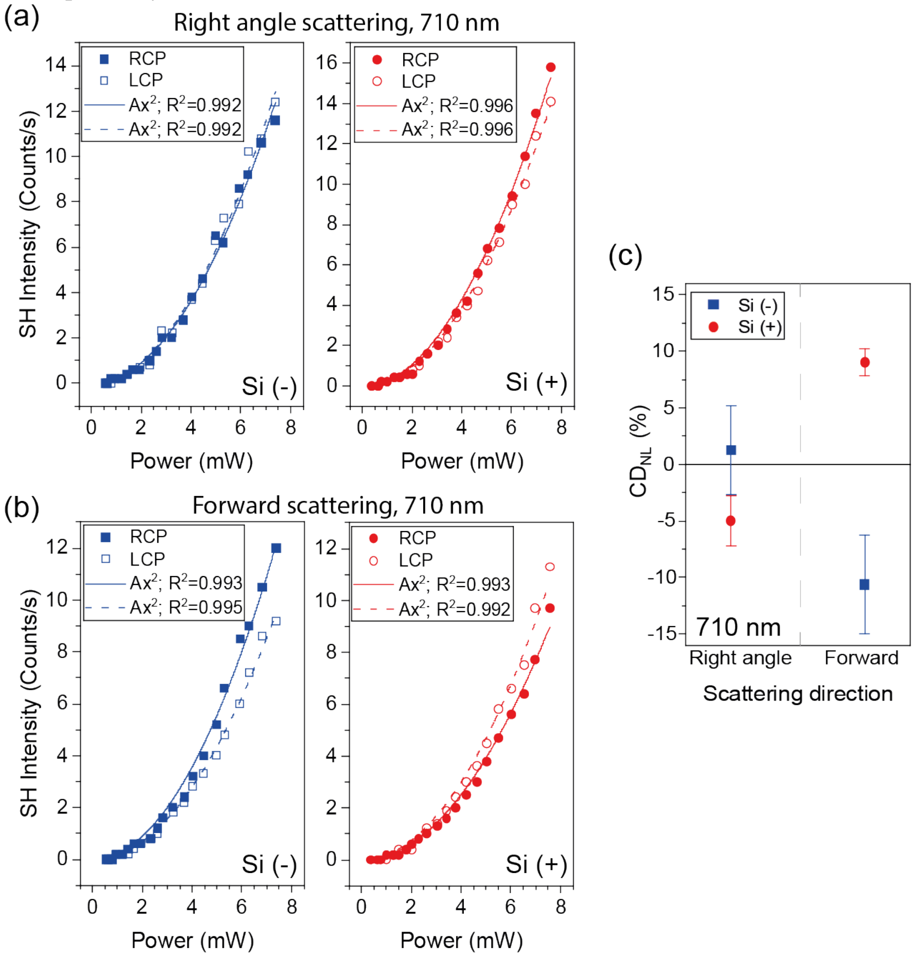

Figure S3: Power dependence measurements for the fundamental wavelength of 710 nm. *Right angle scattering is shown in (a) with Si (-) in the left panel and Si (+) in the right panel. For the Si (-) there are excellent fits to y=Ax², however there is no clear sign of chirality. For the Si (+) we see that RCP has a greater intensity then the LCP, while retaining the square*

dependence. In (b) the same measurements are made in forward scattering. This time the Si (-) in the left panel shows a strong chiral signal, where the RCP light is more intense than the LCP light. For Si (+) in the right panel, the LCP light is more intense than the RCP, showing an inverted chirality to both the Si (-) in forward scattering, and to the right angle scattering for Si (+). In (c) we show the corresponding nonlinear CD spectra for both handedness of the nanohelices in right angle and forward scattering. There is some overlap in the CD in right angle scattering, but in forward scattering there is a clear contrast, which are both opposite in sign to the right angle direction.

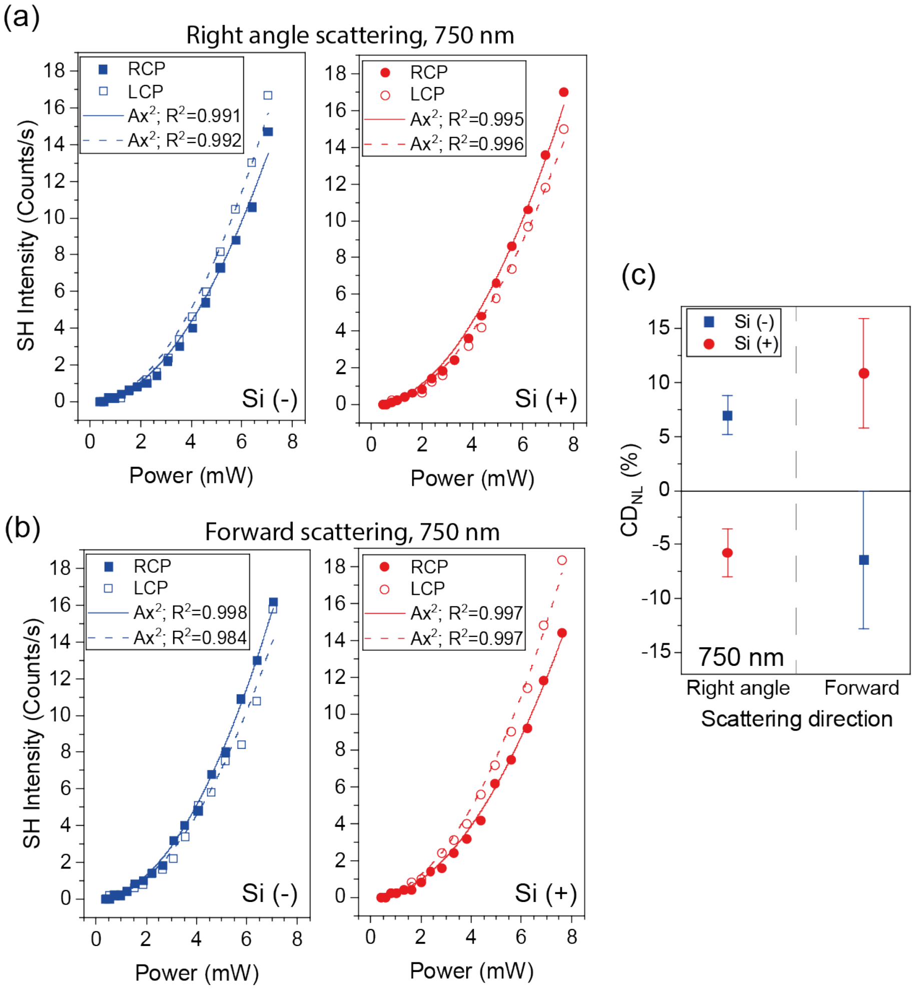

Figure S4: Power dependence measurements for the fundamental wavelength of 750 nm. *(a) shows the right angle scattering for both Si (-) and Si (+) in the left and right panels respectively. In both cases there is an excellent fit to y=Ax², and a definitive sign of chirality. The chirality also inverts when changing from Si (–) to Si (+). In (b), forward scattering power dependence is obtained. Once again, the data are in excellent agreement with a second order nonlinear effect. The chirality changes between the sample and is also the opposite to the right angle scattering in both cases. In (c) we again show the corresponding nonlinear CD where a clear inversion in the sign of the CD is shown between enantiomorphs and in directions.*

We also display the chirality from each power dependence measurement for the three wavelengths in **figure s5** as the nonlinear ellipticity, given by

$$\tan\theta = \frac{180}{\pi}\frac{\sqrt{I_{RCP}^{2\omega}} - \sqrt{I_{LCP}^{2\omega}}}{\sqrt{I_{RCP}^{2\omega}} + \sqrt{I_{LCP}^{2\omega}}}$$

Where θ is the ellipticity, and $I_{RCP}^{2\omega}$ and $I_{LCP}^{2\omega}$ are the intensities of the scattered light when illuminated with right and left circularly polarised light respectively. In (a) we show the ellipticity for right angle scattered light and in (b) we show the ellipticity for forward scattered light. They are inverted in sign compared with the g-factor and nonlinear CD measurements previously shown due to the definition of ellipticity. However they are agreement with those calculations.

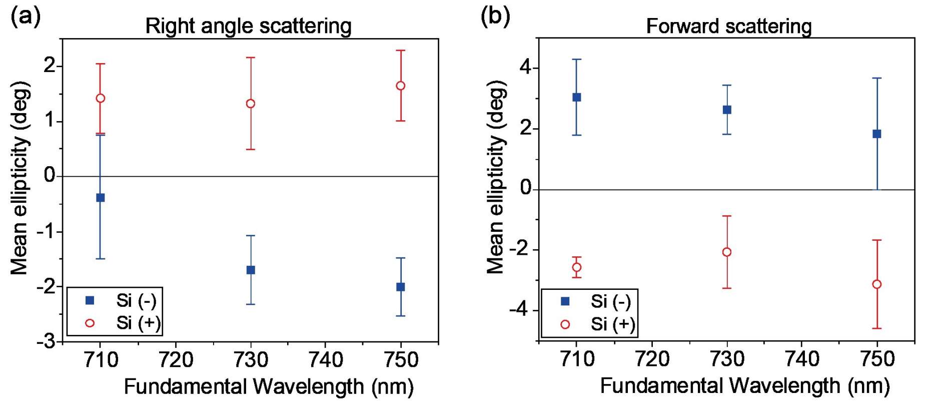

Figure S5: Ellipticity of the Si(-) and SI (+) helices in (a) right angle scattering and (b) forward scattering. *We see a large positive ellipticity for the Si (+) helices in right angle scattering that is clearly distinct from the corresponding ellipticity for Si (-). Likewise we see the same in (b) but the ellitpicities have inverted sign, in agreement with the g-factor and* CD_{NL} *measurements.*

4. General Theory

To fully investigate the effect of scattering angle, on second harmonic scattering in fluid or randomly oriented media, requires the use of intricate tensor rotational averaging methods. Identifying the subtle difference in harmonic scattering intensity that arises for circularly polarized light in chiral media, according to the relative handedness, is an especially demanding calculation since it invokes high ranks. Nonetheless the principles are the same as has been used to elicit the angle dependence of Raman optical activity[7], for example.

As shown many years ago[8] three molecular response tensors are implicated in the circular differential of second harmonic (hyper-Rayleigh) scattering. One is the familiar the molecular hyperpolarizability β, a third rank tensor based on electric dipole (E1) interactions with each of the two input frequency photons, and with the harmonic frequency output photon into which the input is converted. In terms of basic symmetry, the $E1^3$ nature of β gives it odd spatial parity. The interference of β with tensors of even parity can arise only in systems of broken symmetry and is thereby manifested in chirally responsive interactions. There are four tensors of even parity that dominate such effects. Following previously established nomenclature, we write these as ${}^{\alpha}J$, ${}^{\beta}J$, ${}^{\alpha}K$ and ${}^{\beta}K$.

The J tensors are of $E1^2M1$ character, M1 denoting a transition magnetic dipole; the K tensors are $E1^2E2$, where E2 signifies a transition electric quadrupole. In the tensor ${}^{\alpha}J$, magnetic dipole interaction occurs in the harmonic photon output; in ${}^{\beta}J$, this form of interaction is associated with one of the input photons. Equally, ${}^{\alpha}K$ represents harmonic emission from an electric quadrupole interaction, while in ${}^{\beta}K$ the quadrupole coupling occurs with an input photon.

Index symmetry properties play a huge role in simplifying the form of equations for chiral harmonic scattering. However, the form of index symmetry is generally not the kind associated with molecular structural symmetry. In achiral systems, optical response tensors such as the hyperpolarizability will often have fewer independent components than the tensor rank suggests. For example, in systems of threefold rotational symmetry, we have $\beta_{xyz} = \beta_{yxz}$ due to the interchangeability of the x and y axes – an extensive analysis is given in a recent work by Wu and Xiong.[9] However, chiral media seldom have such structural symmetry; in many cases the relevant point group C_1 contains no other than the identity operation. Nonetheless, what we do have is tensor index symmetry resulting from the indistinguishability of the two input photons. In consequence the third rank β and J tensors (in which each of their three indices can be x, y or z) each have not 3^3 independent components, but only 18. Equally the fourth rank K tensors have 54 independent elements.

In principle we might at this stage anticipate chiral harmonic rate equations to be cast in terms of $2(18 \times 18) + 2(18 \times 54) = 2592$ tensor component products. However, the implementation of orientational averaging is to produce results cast in terms of a much more manageable set of their linear combinations. It is important to note that this averaging is performed on the interference terms that arise in the equation for the observable, the harmonic intensity. (This feature of an incoherent optical process is to be contrasted with the averaging of individual quantum amplitude terms within the products, which produces the much stronger signals in coherent harmonic generation, when allowed; see later.)

The linear combinations that arise are weight-0 (i.e. scalar, rotationally invariant) elements of sixth- and seventh-rank tensors formed by the outer products of β with the J and K tensors, respectively. Moreover, these

invariants may be identified by taking the inner products of $\beta \otimes J$ and $\beta \otimes K$ with rank 6 and 7 isotropic tensors.[10]

Two examples may suffice. The outer product of $\beta_{\lambda\mu\nu}\,{}^{\alpha}J_{o\pi\rho}$ has the six free Cartesian indices $\lambda, \mu, \nu, o, \pi, \rho$, and one of several weight-0 invariants is secured from the inner product with an isotropic sixth-rank tensor $\delta_{\lambda\mu}\delta_{\nu o}\delta_{\pi\rho}$, where each δ is a Kronecker delta. In the explicit product $\beta_{\lambda\mu\nu}\,{}^{\alpha}J_{o\pi\rho}\,\delta_{\lambda\mu}\delta_{\nu o}\delta_{\pi\rho}$, each of the six repeated indices is summed over all three Cartesian directions – the 'contraction' being affected by using the Einstein summation convention. In fact, the sixth rank isotropic tensor has fifteen linearly independent isomers, but pair index symmetry properties of β and of ${}^{\alpha}J$ results in just six linearly independent rotational invariants.

Equally, taking the case of $\beta\,{}^{\alpha}K$ interference terms, each such outer product has seven free indices, requiring contraction with one of the seventh rank isotropic tensors, for example $\beta_{\lambda\mu\nu}\,{}^{\alpha}K_{o\pi\rho\sigma}\,\delta_{\lambda\mu}\delta_{\nu o}\varepsilon_{\pi\rho\sigma}$, where ε is the Levi Civita antisymmetric tensor. In this case there are 91 linearly independent isomers of the rank 7 isotropic tensors, but again due to the pair index symmetry in the β and ${}^{\alpha}K$ tensors, many fewer non-vanishing independent rotational invariants arise: just seven in this case.

As can be shown by irreducible tensor methods,[11] the maximum number of rotational invariants that can arise from the βJ and βK products altogether is 31 (six each from $\beta^{\alpha}J$ and $\beta^{\beta}J$, seven from $\beta^{\alpha}K$ and twelve from $\beta^{\beta}K$). In fact, only 25 arise in the explicit rate equations and these are given in Table ST2, where k is the wavenumber of the fundamental frequency pump.

$j_1 = \mathrm{Im}\,\beta_{\lambda\lambda\mu}\,{}^{\alpha}J_{\nu\mu\nu}$	$j_6 = \mathrm{Im}\,\beta_{\lambda\mu\mu}\,{}^{\beta}J_{\nu\lambda\nu}$	$k_3 = k\beta_{\lambda\mu\nu}\,{}^{\alpha}K_{o\pi\nu\mu}\varepsilon_{\lambda o\pi}$	$k_8 = k\beta_{\lambda\mu\mu}\,{}^{\beta}K_{o\pi\rho\rho}\varepsilon_{\lambda o\pi}$	$k_{13} = k\beta_{\lambda\mu\nu}\,{}^{\beta}K_{o\pi\nu o}\varepsilon_{\lambda\mu\pi}$
$j_2 = \mathrm{Im}\,\beta_{\lambda\mu\nu}\,{}^{\alpha}J_{\mu\lambda\nu}$	$j_7 = \mathrm{Im}\,\beta_{\lambda\mu\nu}\,{}^{\beta}J_{\lambda\mu\nu}$	$k_4 = k\beta_{\lambda\mu\nu}\,{}^{\alpha}K_{o\pi\pi\nu}\varepsilon_{\lambda\mu o}$	$k_9 = k\beta_{\lambda\mu\nu}\,{}^{\beta}K_{o\nu o\sigma}\varepsilon_{\lambda\mu\sigma}$	$k_{14} = k\beta_{\lambda\mu\nu}\,{}^{\beta}K_{o\pi\nu\mu}\varepsilon_{\lambda o\pi}$
$j_3 = \mathrm{Im}\,\beta_{\lambda\lambda\mu}\,{}^{\beta}J_{\mu\nu\nu}$	$j_8 = \mathrm{Im}\,\beta_{\lambda\mu\nu}\,{}^{\beta}J_{\mu\lambda\nu}$	$k_5 = k\beta_{\lambda\mu\nu}\,{}^{\alpha}K_{\nu\pi\rho\rho}\varepsilon_{\lambda\mu\pi}$	$k_{10} = k\beta_{\lambda\mu\nu}\,{}^{\beta}K_{o\nu\mu\sigma}\varepsilon_{\lambda o\sigma}$	$k_{15} = k\beta_{\lambda\mu\nu}\,{}^{\beta}K_{o\pi\pi\nu}\varepsilon_{\lambda\mu o}$
$j_4 = \mathrm{Im}\,\beta_{\lambda\lambda\mu}\,{}^{\beta}J_{\nu\mu\nu}$	$k_1 = k\beta_{\lambda\mu\mu}\,{}^{\alpha}K_{o\pi\rho\rho}\varepsilon_{\lambda o\pi}$	$k_6 = k\beta_{\lambda\mu\mu}\,{}^{\beta}K_{o\pi o\sigma}\varepsilon_{\lambda\pi\sigma}$	$k_{11} = k\beta_{\lambda\mu\nu}\,{}^{\beta}K_{o\nu\rho\rho}\varepsilon_{\lambda\mu o}$	$k_{16} = k\beta_{\lambda\mu\nu}\,{}^{\beta}K_{\nu\pi\pi\sigma}\varepsilon_{\lambda\mu\sigma}$
$j_5 = \mathrm{Im}\,\beta_{\lambda\mu\mu}\,{}^{\beta}J_{\lambda\nu\nu}$	$k_2 = k\beta_{\lambda\mu\nu}\,{}^{\alpha}K_{o\nu\rho\rho}\varepsilon_{\lambda\mu o}$	$k_7 = k\beta_{\lambda\mu\mu}\,{}^{\beta}K_{o\pi\pi\sigma}\varepsilon_{\lambda o\sigma}$	$k_{12} = k\beta_{\lambda\mu\nu}\,{}^{\beta}K_{o\pi o\nu}\varepsilon_{\lambda\mu\pi}$	$k_{17} = k\beta_{\lambda\mu\nu}\,{}^{\alpha}K_{\nu\pi\rho\rho}\varepsilon_{\lambda\mu\pi}$

Table ST2: Linearly independent molecular invariants responsible for circular differential second harmonic scattering.

This full set subsumes 14 that arise from βK products, featured in the results for twisted light recently identified by Forbes.[12] Each of the molecular response parameters represents a different sum of products of components of β with components of the J and K tensors, as for example this explicit case:

$$\begin{aligned} j_5 = \mathrm{Im}\Big(&\beta_{xxx}\,{}^{\beta}J_{xxx} + \beta_{xxx}\,{}^{\beta}J_{xyy} + \beta_{xxx}\,{}^{\beta}J_{xzz} + \beta_{xyy}\,{}^{\beta}J_{xxx} + \beta_{xyy}\,{}^{\beta}J_{xyy} + \beta_{xyy}\,{}^{\beta}J_{xzz} \\ &+\beta_{xzz}\,{}^{\beta}J_{xxx} + \beta_{xzz}\,{}^{\beta}J_{xyy} + \beta_{xzz}\,{}^{\beta}J_{xzz} + \beta_{yxx}\,{}^{\beta}J_{yxx} + \beta_{yxx}\,{}^{\beta}J_{yyy} + \beta_{yxx}\,{}^{\beta}J_{yzz} + \beta_{yyy}\,{}^{\beta}J_{yxx} \\ &+\beta_{yyy}\,{}^{\beta}J_{yyy} + \beta_{yyy}\,{}^{\beta}J_{yzz} + \beta_{yzz}\,{}^{\beta}J_{yxx} + \beta_{yzz}\,{}^{\beta}J_{yyy} + \beta_{yzz}\,{}^{\beta}J_{yzz} + \beta_{zxx}\,{}^{\beta}J_{zxx} + \beta_{zxx}\,{}^{\beta}J_{zyy} \\ &+\beta_{zxx}\,{}^{\beta}J_{zzz} + \beta_{zyy}\,{}^{\beta}J_{zxx} + \beta_{zyy}\,{}^{\beta}J_{zyy} + \beta_{zyy}\,{}^{\beta}J_{zzz} + \beta_{zzz}\,{}^{\beta}J_{zxx} + \beta_{zzz}\,{}^{\beta}J_{zyy} + \beta_{zzz}\,{}^{\beta}J_{zzz}\Big) \end{aligned}$$

The entirety of the general result for a chirality-sensitive difference in the harmonic intensity, emitted at an arbitrary angle with respect to the input beam, is expressible in terms of the j and k coefficients as follows:

$$\begin{aligned} \Delta I^{\mathrm{R\text{-}L}} =& \left(-16j_3 + 24j_4 + 20j_5 - 16j_6 + 44j_7 - 24j_8 + 8k_6 - 10k_7 + 12k_8 - 20k_9 + 10k_{10} \right. \\ & \left. +4k_{12} + 36k_{13} - 20k_{14} - 8k_{15} + 12k_{16} - 16k_{17}\right) \\ & +\left(-28j_1 + 28j_2 + 16k_1 - 24k_2 - 24k_3 + 16k_4 - 48k_5\right)\cos\theta \\ & +\left(24j_3 - 36j_4 - 16j_5 + 24j_6 + 24j_7 - 36j_8 + 16k_6 - 13k_7 + 10k_8 - 26k_9 + 13k_{10} + 28k_{11} \right. \\ & \left. +22k_{12} - 26k_{13} + 2k_{14} - 16k_{15} + 10k_{16} - 4k_{17}\right)\cos^2\theta \\ & +\left(-20k_1 + 12k_2 + 12k_3 + 20k_4 - 60k_5\right)\cos^3\theta \end{aligned} \qquad (1)$$

where θ is defined as $\cos^{-1}\left(-\hat{\mathbf{k}}.\hat{\mathbf{k}}'\right)$, with $\hat{\mathbf{k}}$, $\hat{\mathbf{k}}'$ the unit propagation vectors for the input pump and detected harmonic, respectively. Note that coherent second harmonic generation in the forward direction is forbidden in any isotropic fluid – as indeed is the coherent generation of any harmonic using circularly polarized light (save for under the highly intense conditions that produce high-order harmonics).[13] Hence the second harmonic signal

detected at any angle, in the cases studied here, can only result from the incoherent form of interaction that generates the above result. The explicit results for forward and for right-angled scattering are as follows:

$$\begin{aligned}\Delta I_{\text{fwd}}^{\text{R-L}} &= 28j_1 - 28j_2 + 8j_3 - 12j_4 + 4j_5 + 8j_6 + 68j_7 - 60j_8 + 4k_1 + 12k_2 + 12k_3 - 36k_4 \\ &+ 108k_5 + 24k_6 - 23k_7 + 22k_8 - 46k_9 + 23k_{10} + 28k_{11} + 26k_{12} + 10k_{13} - 18k_{14} - 24k_{15} \\ &+ 22k_{16} - 20k_{17}\end{aligned} \quad (2)$$

$$\begin{aligned}\Delta I_{\text{rt.}\angle}^{\text{R-L}} &= -16j_3 + 24j_4 + 20j_5 - 16j_6 + 44j_7 - 24j_8 + 8k_6 - 10k_7 + 12k_8 - 20k_9 + 10k_{10} \\ &+ 4k_{12} + 36k_{13} - 20k_{14} - 8k_{15} + 12k_{16} - 16k_{17}\end{aligned} \quad (3)$$

The orders of magnitude of each molecular invariant might be anticipated to be broadly similar, but there is absolutely no basis for supposing them to have the same sign. Accordingly, the differently weighted linear combinations in equations (2) and (3) cannot be expected to display any correlation. In particular, the sign of the circular intensity differential in the harmonic scattering at any angle cannot be interpreted as indicating a specific handedness of the scatterer.

In principle, the analysis of results secured over a range of scattering angles enables the relative values for the four bracketed terms in equation (1) – i.e., the coefficients of $\cos^n(\theta)$ with $n = 0\ldots3$ – to be independently ascertained. The most rapidly varying term, the coefficient of $\cos^3(\theta)$, carries the information that is most directly interpretable in terms of physical mechanism, since it arises purely from an interference of electric dipole and quadrupole coupling with the fundamental beam.

5. Numerical simulations

To simulate the linear optical activity of Si nanohelices, we calculated absorption and scattering cross-section (σ_{abs} and σ_{sc}) for LCP and RCP excitations for each orientation, and then calculated the average cross-sections for a random orientation. The extinction cross-section σ_{ext} is then calculated as the sum of the average scattering and absorption cross-sections (σ_{sc} and σ_{abs}, respectively), and the linear CD as: $CD_{ext}[\%] = 100(\sigma_{ext,LCP} - \sigma_{ext,RCP})/(\sigma_{ext,LCP} + \sigma_{ext,RCP})$, as shown in Figure 1(e) in the main manuscript. In Figure S5 we show the contributions of each orientation to the CD signal of Si(-) helix.

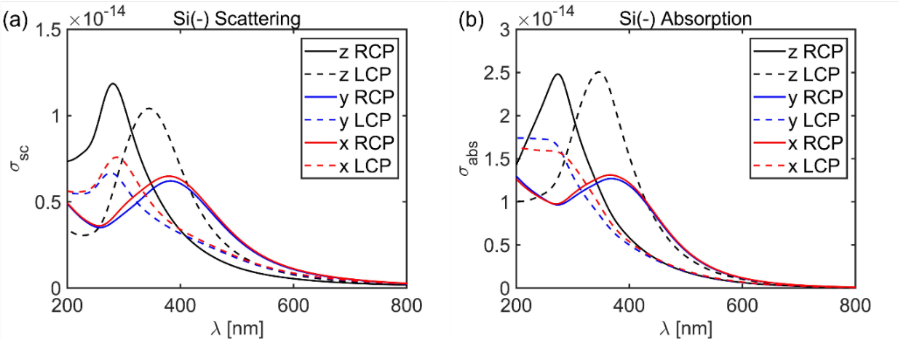

Figure S5: *(a) Scattering and (b) absorption cross-section [m²] for Si(-) helices of different orientations, excited in water under normal incidence with RCP or LCP polarization.*

To compare the linear absorption CD of the z-oriented helix to the vertically aligned helices on the substrate, we plot linear absorption CD as: $CD_{abs}[\%] = 100(\sigma_{abs,LCP} - \sigma_{abs,RCP})/(\sigma_{abs,LCP} + \sigma_{abs,RCP})$, Figure S6.

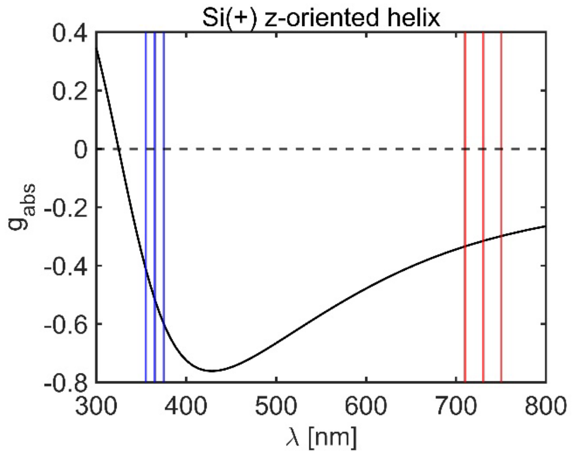

Figure S6: *Absorption CD for z-oriented Si(+) nanohelix is in agreement with linear CD of vertical NW ensemble investigated in ref. [48] in the main manuscript. Vertical lines correspond to fundamental (red) and second harmonic (blue) wavelengths in the nonlinear experiment.*

In Figure S7, we plot the 3D distribution of the absorption density in z- and x-oriented Si(+) nanohelices for all fundamental wavelengths. As expected from Figure S6, z-oriented nanohelix couples better with RCP polarization, while the chirality inverts for x-oriented nanohelix. At all fundamental wavelengths, there is a noticeable difference in absorption between coupling with LCP and RCP incident beam.

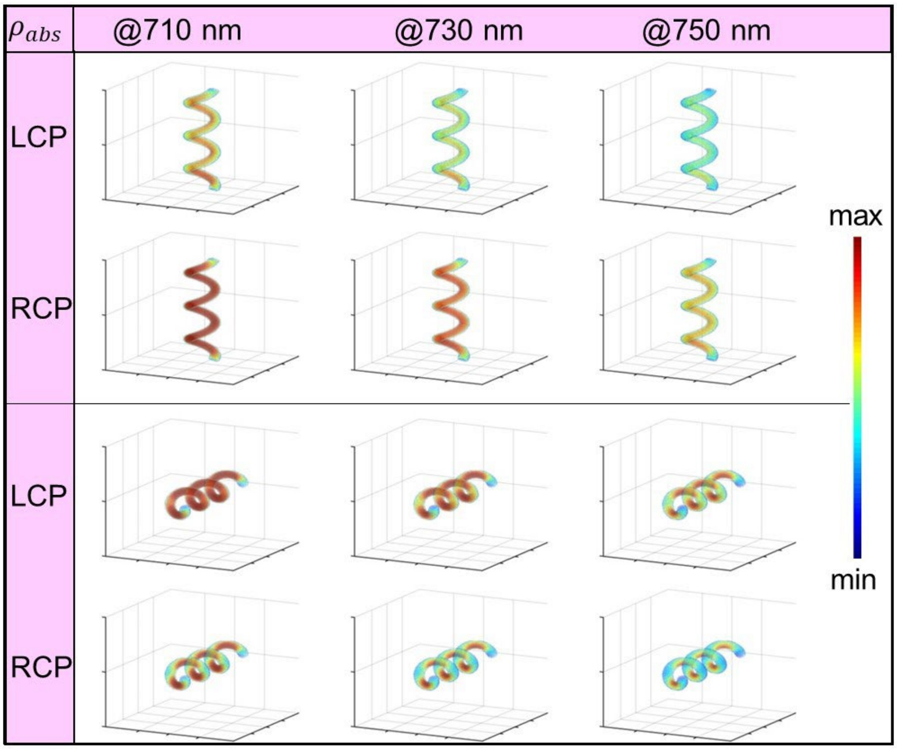

Figure S7: *3D distribution of the absorption density for Si(+) helix excited from the top at wavelengths of our fundamental beams, having RCP or LCP polarizations.*

In the following, we investigate radiation patterns of the scattering for three Si(+) nanohelix orientations at 730 nm, Figure S8. The light is incident in the negative z-direction, which is the direction of the "forward" scattering in the experiment; x-direction corresponds to the "right angle" scattering in the experiment. We notice that the radiation patterns for both excitations correspond to Rayleigh rather than Mie-type scattering. As expected, they are dependent on the helix orientation with respect to the light direction and handedness, and that z-oriented nanohelix strongly scatters RCP light, opposite to the x- and y-oriented nanohelices.

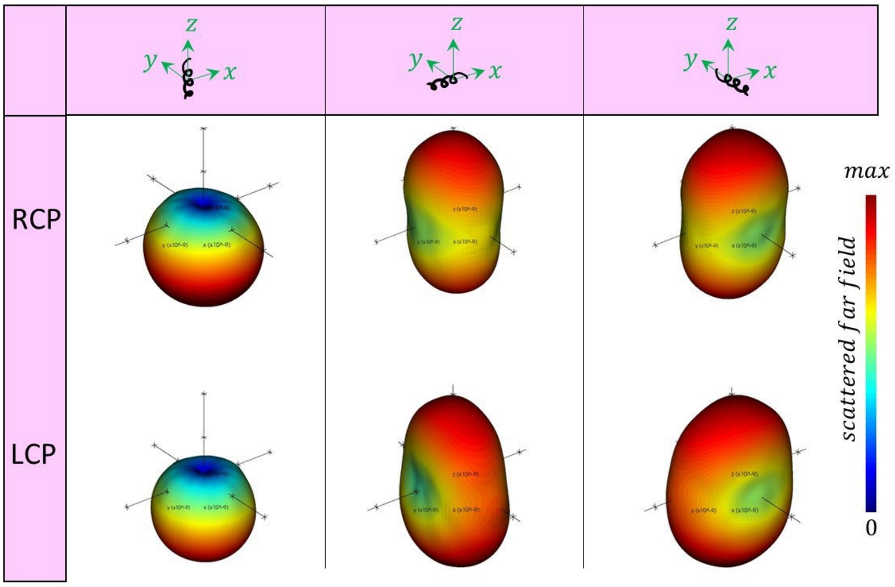

***Figure S8**: Far-field scattering radiation pattern when z- (left), x- (middle) and y-oriented (right) Si (+) nanohelix is excited with RCP or LCP at 730 nm.*

We next show XY and XZ 2D cuts of this field, averaged over the random nanohelix orientation. In Figure S9, we show the 2D cuts of the scattered radiation for different orientations of Si (+) helices, excited from the top by RCP or LCP light at 730 nm. As previously seen, x- and y-oriented helices strongly scatter LCP light in both right and forward direction, while z-oriented helices show opposite behaviour. However, scattering averaged on all orientations is stronger for LCP in both forward and right-angle directions, contrary to the inverted sign of the nonlinear experiment (Figure 3c-d in the main manuscript); this trend is seen at all fundamental wavelengths, Figure S10.

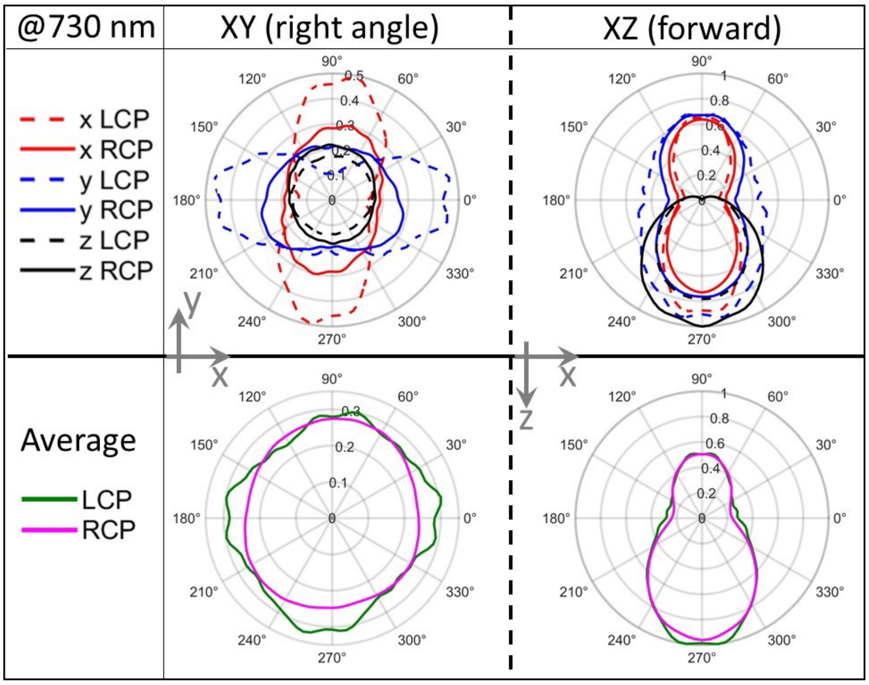

***Figure S9**: Polar plots of the linear scattering at 730 nm in the far-field planes; top: xy and xz plane section of the scattering for all orientations of helices, normalized to the maximum scattering of the z-oriented helix excited with RCP; bottom: averaged scattering for LCP and RCP excitations, normalized to the maximum scattering in the xz-plane under LCP excitation. The overall scattering for a randomly oriented helix is greater for the LCP excitations in all directions. Therefore, chirality in the linear case does not invert sign the right and forward scattering, unlike in the nonlinear experiment.*

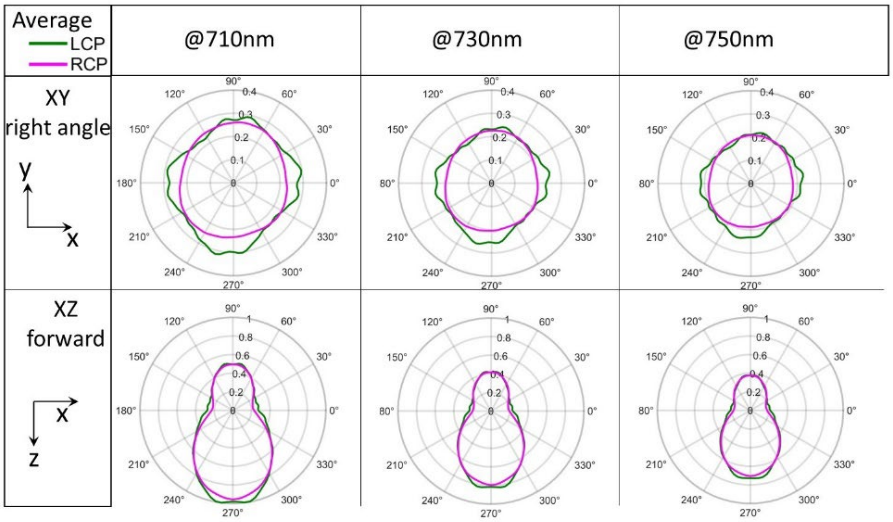

***Figure S10**: Averaged polar plots of the linear scattering for a randomly oriented Si(+) nanohelix at fundamental wavelengths; all plots are normalized to the maximum forward scattering, for LCP excitation, at 710 nm.*

Different scattering and absorption contributions of each orientation led to the good agreement of the numerical model with the experimentally measured linear CD. Next, we investigate the contributions of different multipoles to the observed scattering. To this end, we extract wavelength-dependent distributions of electric field from 3D field monitors, and refractive index distributions for the nanohelix from Lumerical, and import it into MENP,[7] an open-source MATLAB-based solver; MENP then calculates the displacement current distributions and resolves contributions of the electric dipole (ED), magnetic dipole (MD), electric quadrupole (EQ) and magnetic quadrupole (MQ). In Figure 4 we show spectra of these contribution for z- and x-oriented nanohelices. All the dominant modes are resonant around second harmonic wavelengths, and their contribution strongly depends on the orientation of the helix. Notably, z-oriented helix has dominant magnetic dipole mode, while the contribution of electric dipole and electric quadrupole modes are not negligible. In Table ST1 we calculate the percentage of the contribution of each mode to the overall scattering at 365 nm. Note that there can be differences with respect to the overall scattering cross-section shown in Figure 4a in the main manuscript, due to the expansion which takes into account only four multipole modes, and due to the differences in the monitors surrounding the single nanohelix.

@365nm [%]	X-oriented LCP	X-oriented RCP	Z-oriented LCP	Z-oriented RCP
Electric dipole	75	84	20	23
Magnetic dipole	13	4	56	52
Electric quadrupole	11	2	19	18
Magnetic quadrupole	1	10	5	7

Table ST1: Contribution of electric dipole, magnetic dipole, electric quadrupole and magnetic quadrupole to the overall scattering at 365 nm; it strongly depends on the excitation handedness and direction with respect to the helix.

[1] Evlyukhin, A. B. et al. Optical Response Features of Si-Nanoparticle Arrays, *Phys. Rev. B,* **2010**, 82, 045404.
[2] Gómez-Medina, R. et. al. Electric and Magnetic Dipolar Response of Germanium Nanospheres: Interference Effects, Scattering Anisotropy, and Optical Forces, *J. Nanophotonics,* **2011** (5), 053512. DOI: 10.1117/1.3603941.
[3] Sigalas, M. M. et al. Electric field enhancement between two Si microdisks, *Opt. Express,* **2007**, 15, 14711-14716.
[4] Shibanuma, T. et al. Unidirectional light scattering with high efficiency at optical frequencies based on low-loss dielectric nanoantennas, *Nanoscale,* **2016**, 8, 14184-141.
[5] Kuznetsov, A. et al. Magnetic light, *Sci. Rep.* **2012**, 2, 492.
[6] Bakker, R. M. et al. Magnetic and electric hotspots with silicon nanodimers, *Nano Lett.* **2015**, 15, 3, 2137–2142.

[7] Andrews, D. L. Rayleigh and Raman optical activity: An analysis of the dependence on scattering angle*, J. Chem. Phys*, 1980, 72, (7), 4141-4144, DOI: 10.1063/1.439643.
[8] Andrews, D. L. Thirunamachandran, T. Hyper−Raman scattering by chiral molecules, *J. Chem. Phys*, **1979**, 70, pp 1027-1030.
[9] Wu, Z. Xiong, W. Neumann's principle based eigenvector approach for deriving non-vanishing tensor elements for nonlinear optics, *J. Chem. Phys*, **2022**, 157, 134702.
[10] Jeffreys, H. On isotropic tensors, *Proc. Camb. Phil. Soc.*, **1973**, 73, pp 173-176.
[11] Andrews, D. L. Symmetry-based identification and enumeration of independent tensor properties in nonlinear and chiral optics, *J. Chem. Phys.* **2023**, 158, (3), 034101 DOI: 10.1063/5.0129636.
[12] Forbes, K. A. Nonlinear chiral molecular photonics using twisted light: hyper-Rayleigh and hyper-Raman optical activity, *J. Opt.*, **2020**, 22, (9), 095401. DOI: 10.1088/2040-8986/aba0fd
[13] Alon, O. E. Averbukh, V. Moiseyev, N. Selection Rules for High Harmonic Generation Spectra, *Phys. Rev. Lett.*, **1998**, 80, (17), 3743-3346. DOI: 10.1103/PhysRevLett.80.3743
[14] Hinamoto, T. Fujii, M. MENP: An Open-Source MATLAB Implementation of Multipole Expansion for Nanophotonics, *OSA Continuum*, **2021**, 4, (5), 1640-1648. DOI: 10.1364/OSAC.425189.
